# Characterizing intergenic transcription at RNA polymerase II binding sites in normal and cancer tissues

**DOI:** 10.1016/j.xgen.2023.100411

**Published:** 2023-09-29

**Authors:** Pierre de Langen, Fayrouz Hammal, Elise Guéret, Jean-Christophe Mouren, Lionel Spinelli, Benoit Ballester

**Affiliations:** 1 Aix Marseille Univ, INSERM, TAGC, Marseille, France

**Keywords:** enhancers, non-coding DNA, regulatory genomics, intergenic, RNA Polymerase II, gene regulation, cancer genomics, non-coding transcription

## Abstract

Intergenic transcription in normal and cancerous tissues is pervasive but incompletely understood. To investigate this, we constructed an atlas of over 180,000 consensus RNA polymerase II (RNAPII)-bound intergenic regions from 900 RNAPII chromatin immunoprecipitation sequencing (ChIP-seq) experiments in normal and cancer samples. Through unsupervised analysis, we identified 51 RNAPII consensus clusters, many of which mapped to specific biotypes and revealed tissue-specific regulatory signatures. We developed a meta-clustering methodology to integrate our RNAPII atlas with active transcription across 28,797 RNA sequencing (RNA-seq) samples from The Cancer Genome Atlas (TCGA), Genotype-Tissue Expression (GTEx), and Encyclopedia of DNA Elements (ENCODE). This analysis revealed strong tissue- and disease-specific interconnections between RNAPII occupancy and transcriptional activity. We demonstrate that intergenic transcription at RNAPII-bound regions is a novel per-cancer and pan-cancer biomarker. This biomarker displays genomic and clinically relevant characteristics, distinguishing cancer subtypes and linking to overall survival. Our results demonstrate the effectiveness of coherent data integration to uncover intergenic transcriptional activity in normal and cancer tissues.

## Highlights

• An RNAPII atlas of intergenic transcription in normal tissues and cancer samples
• Intergenic atlas shows enhancer-like characteristics and transcriptional signals
• Meta-clustering reveals shared transcriptional patterns among tissues and cancer types
• Identified intergenic markers associated with cancer genes and survival

## Graphical abstract

**Figure:**
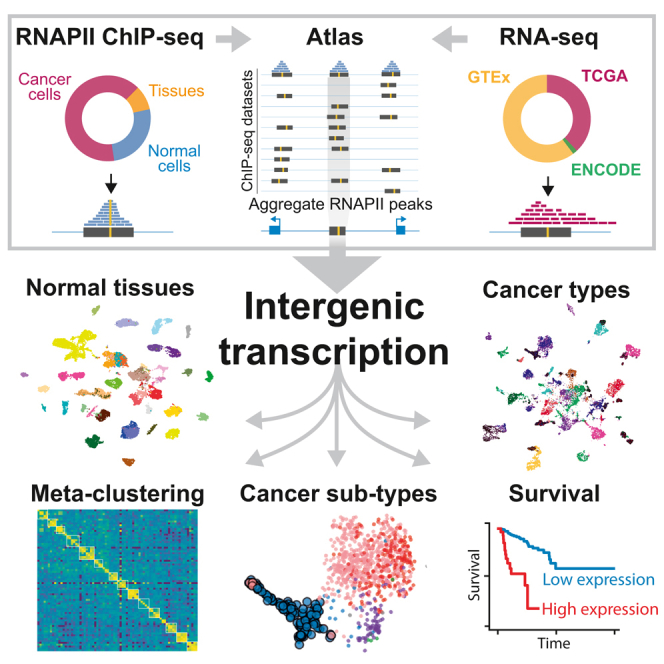

## Teaser

We developed an atlas of intergenic transcription using RNAPII binding sites to connect genomic and transcriptomic data in normal tissues and cancer samples. The atlas enables investigation of tissue specificity and core regulatory elements. Meta-clustering reveals shared transcription patterns among tissues and cancer types. We identified intergenic markers that are associated with known cancer genes and predictive of overall survival. Our study demonstrates the effectiveness of integrating diverse public datasets to characterize intergenic transcription in normal and cancer tissues, addressing limitations of previous techniques.

## Introduction

Transcription is a fundamental process in biology that transcribes DNA into biologically active and cell-type-specific RNA molecules. The majority of transcription is carried out by RNA polymerase II (RNAPII), which generates mRNAs that are subsequently translated into proteins. However, intergenic active regions have been shown to cover a much larger fraction of the genome than expected.^1^ Indeed, RNAPII transcribes a wide variety of intergenic active regions, such as different types of non-coding RNAs (ncRNAs)^2^ or enhancer RNAs (eRNAs) that have been found to be major sites of intergenic transcription.^3^

While genes and their protein products have been the main interest in basic and cancer research, an increasing amount of genomic data support the biological and clinical relevance of intergenic transcription. Aberrant expression of ncRNAs has been found in cancer^4^ and non-cancer disease,^5^ and a vast majority of trait or disease-associated variants lie in non-coding regions of the genome.^6^ Despite significant progress in describing enhancer transcription,^3^^,^^7^^,^^8^^,^^9^^,^^10^ efforts to fully identify intergenic transcription remain a challenge. This is primarily due to a limited amount of sequencing assays like global run on sequencing (GRO-seq)^11^ or its derivatives,^12^^,^^13^ impacting the discovery of a broader intergenic transcription landscape.

In this study, we compiled each available RNAPII chromatin immunoprecipitation sequencing (ChIP-seq) dataset from the GEO^14^ and Encyclopedia of DNA Elements (ENCODE)^1^ to construct an atlas of RNAPII-bound intergenic regions in the human genome. Our approach, which targets RNAPII binding rather than the resulting ncRNA, aims to minimize the limitations of RNA abundance and stability. This approach enables exploration of active intergenic regions in a broad range of cell types and tissues, which have not been extensively studied before.

We hypothesize that intergenic RNAPII-bound regions of significance exhibit a biotype-specific signature, reflected in biotype-specific RNA sequencing (RNA-seq) expression across resources such as the Genotype-Tissue Expression (GTEx^15^), The Cancer Genome Atlas (TCGA^16^) and The Encyclopedia of DNA Elements (ENCODE^1^). In this study, we describe tissue-specific bindings by creating an atlas of intergenic RNAPII-bound regions. By analyzing the expression patterns of 28,797 RNA sequencing samples, we identify intergenic transcription on RNAPII-bound regions as a powerful indicator for characterizing tissue types. We show that using intergenic transcription on RNAPII-bound regions results in robust classification of cancer types and subtypes.

Taken together, our study indicates that intergenic transcription at RNAPII binding sites is a powerful indicator for characterizing normal and cancer tissues at the subtype level. While the functional significance of intergenic regions remains an open question, our findings could significantly enhance our understanding of the regulatory programs and clinical relevance of non-coding transcription in various cancers.

## Results

### An atlas of intergenic RNAPII occupancy

**Figure 1:**
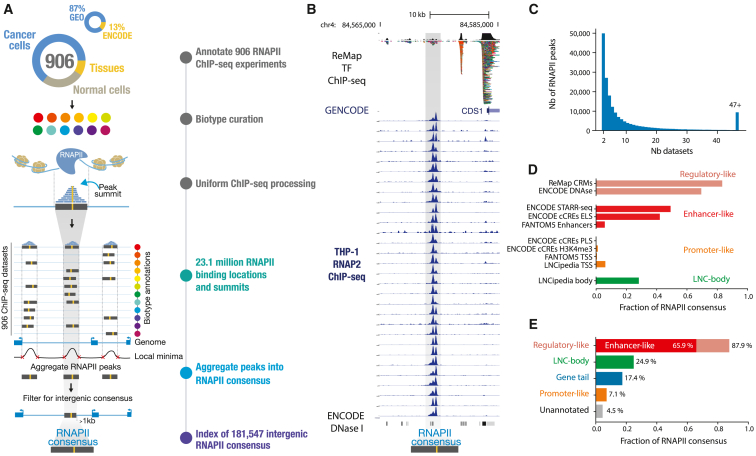
An atlas of intergenic RNAPII occupancy (A) Overview of the RNAPII atlas pipeline; 23.1 million RNAPII-bound regions aggregated across 906 individual datasets jointly identify 181,547 intergenic RNAPII consensus. (B) Genomic example on chromosome 4, showing RNAPII raw ChIP-seq signals across THP-1 cell lines (leukemia, in blue) at the location of a RNAPII consensus (gray bar), with ReMap TP ChIP-seq and ENCODE DNase I tracks. (C) Distribution of the number of datasets across which RNAPII peaks are shared. (D) Comparison of RNAPII consensus location with genomic resources of regulatory and non-coding elements; resources are grouped and colored by genomic characteristics. (E) Annotation of the RNAPII atlas according to genomic characteristics: regulatory like, enhancer like, long non-coding body, gene tail, promoter like, and unannotated.

To create an atlas of intergenic RNAPII binding in the human genome, we collected all available ChIP-seq data targeting RNAPII on a wide variety of cells and tissue biosamples from public biological data warehouses^1^^,^^14^ (Figure 1A). The created atlas aggregates 87% of non-ENCODE datasets and 13% of ENCODE datasets (Figure 1A). This was accomplished through standardized manual curation of sample metadata, uniform biosample annotation, and consistent data processing and quality screening, initiated from the raw sequencing files using the ReMap pipeline (STAR Methods). We conservatively retained 906 RNAPII datasets from diverse cell or tissue types, utilizing various antibodies targeting the POLR2A subunit (Figure S1A). These datasets encompassed a wide range of samples, including cancer cell lines (64%) and “normal” cell lines/tissues (36%) (Figure 1A; STAR Methods). In this study, we focused specifically on intergenic RNAPII-bound regions, preventing us from detecting alternative promoters or any transcriptional events occurring within gene bodies (STAR Methods). We defined intergenic regions as all regions of the genome, excluding all GENCODE transcripts (as well as known long ncRNAs [lncRNAs]) extended by 1 kb at the transcription start site (TSS) and transcription end site (TES) and excluding ENCODE blacklisted regions.^17^ We identified a total of 23,101,589 RNAPII binding events across all 906 datasets, of which 2,525,886 (11.1%) are localized within intergenic regions (averaging 2,787 intergenic binding events per dataset; Figure S1B). A large fraction of RNAPII intergenic binding events (91.7%) is shared across at least two ChIP-seq datasets, suggesting similar occupancy patterns across experiments (Figures 1B and S2). These binding events are also found to be located on clusters of transcription factor ChIP-seq peaks. We developed an aggregative approach to identify across experiments what we refer to as “consensus peaks” (Figures 1A and S3; STAR Methods). By applying this approach, we created an atlas of 181,547 intergenic RNAPII consensus peaks, describing distinct genomic elements bound by RNAPII across multiple biosamples. Our atlas of intergenic RNAPII-bound regions, available on Zenodo,^18^ is based on consensus peaks derived from an average of 13 datasets (Figure 1C), with each consensus having an average width of 410 bp (Figure S1C). Each peak and dataset in the ChIP-seq data contributing to a representative RNAPII consensus can be traced back to its corresponding biosample or cell type category (Figures S1D and S3). We evaluated our created atlas against reference databases of regulatory and non-coding genomic elements^19^^,^^20^^,^^21^^,^^22^^,^^23^ (Figures 1D, 1E, S4–, and S6). We found that the majority of RNAPII consensus peaks (87.9%) were categorized as regulatory regions, with 65.9% showing an enhancer signature (Figure S4A). Furthermore, we observed a concentration of RNAPII consensus downstream of genes (17.4%), within the +1- to +9-kb range (Figures S5A and S5B). Interestingly, these regions exhibit characteristics of regulatory elements and show a strong enrichment of CTCF and CTCFL (BORIS) motifs (Figure S5C). Our findings indicate that the atlas of intergenic RNAPII consensus peaks is predominantly located over regulatory elements and potentially transcribed enhancer regions.

### A normalized vocabulary captures biotype-specific intergenic RNAPII binding

**Figure 2:**
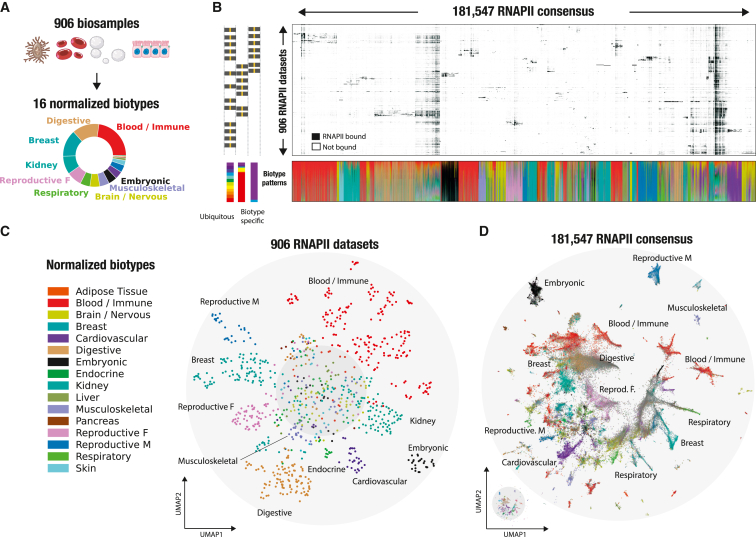
A normalized vocabulary captures biotype-specific intergenic RNAPII binding (A) Distinct tissues and cell lines across 906 biosamples normalized into 16 biotypes. (B) Intergenic RNAPII occupancy in 181,547 consensus regions across 906 biosamples displayed in a visually compressed matrix. The color code used for each RNAPII consensus region corresponds to the biosample tissue of origin, with examples representing either biotype-specific or ubiquitous signatures. This color scheme is consistently applied across all RNAPII consensus regions. Bottom: normalized contribution of a biotype, in terms of peaks, to each RNAPII consensus (STAR Methods). (C) Two-dimensional uniform manifold approximation and projection (UMAP) of all 906 RNAPII ChIP-seq datasets across intergenic RNAPII space, colored by normalized biotype. (D) UMAP representation of all intergenic RNAPII consensus organized by their binding patterns, colored by dominant biotype (STAR Methods; full UMAP available in Zenodo).

The RNAPII atlas covers a significant fraction of the human biological spectrum, including over 203 distinct tissues and cell lines (Figure 2A; Table S1). To facilitate biological interpretation, we grouped biosample annotations based on their tissue of origin or similarity. We then further categorized similar tissues into 16 distinct biotypes to obtain a concise but meaningful high-level annotation of our samples (Table S1; STAR Methods). To simplify genomic interoperability across large resources, the compendium of tissues and cell lines was harmonized using Genotype-Tissue Expression (GTEx), The Cancer Genome Atlas (TCGA), ENCODE biosample nomenclature, as well as cell ontologies.^24^ This results in the RNAPII consensus exhibiting a biological context ranging from biotype-specific to ubiquitous signatures (Figure 2B). Because intergenic RNAPII binding appears to be shared extensively across biosamples (Figures 1B and S2), we aimed to visualize RNAPII occupancy patterns across biosamples and consensus by employing a hierarchical clustering approach (Figure 2B; STAR Methods). Patterns of RNAPII binding were structured into mostly biotype-specific and a few ubiquitous occupancy clusters. We observed what seemed to be a sparse distribution in the intergenic RNAPII atlas, but upon further investigation, we identified diverse and intricate binding patterns. To analyze these patterns, we utilized an unsupervised dimensionality reduction technique (uniform manifold approximation and projection [UMAP]^25^) on the 906 biosamples (Figure 2C) and more than 180,000 RNAPII consensus peaks (Figure 2D). The UMAP visualization across 906 ChIP-seq datasets revealed organized intergenic occupancy patterns across similar biotypes (Figure 2C). Based on their intergenic occupancy patterns, ChIP-seq datasets having similar biotypes of origin were clustered together, while the center of the plot contained datasets with ubiquitous biotype signatures. For example, ChIP-seq datasets for digestive biosamples (represented by brown dots, n = 126 samples) were predominantly clustered together, suggesting that intergenic RNAPII occupancy is representative of the sample biology but also that the biosample curation is coherent. Next, we visualized the 181,547 intergenic RNAPII consensus peaks according to their binding patterns and biotype labels (Figure 2D; STAR Methods). To facilitate biological interpretation of an RNAPII consensus, each consensus was labeled with its most frequent biotype or labeled in gray when ubiquitous. By visualizing the intergenic RNAPII atlas, we were able to identify distinct occupancy patterns that are specific to certain biotypes. This framework was also applied to 890 H3K27ac datasets, successfully demonstrating its ability to identify biotype-specific clusters of histone modifications (Figure S7). The RNAPII atlas, generated by leveraging 906 ChIP-seq datasets, provides a valuable biotype-specific summary of intergenic RNAPII binding. Its potential to uncover intergenic transcriptional activities makes this atlas an innovative tool.

### Revealing tissue-specific regulatory signatures

**Figure 3:**
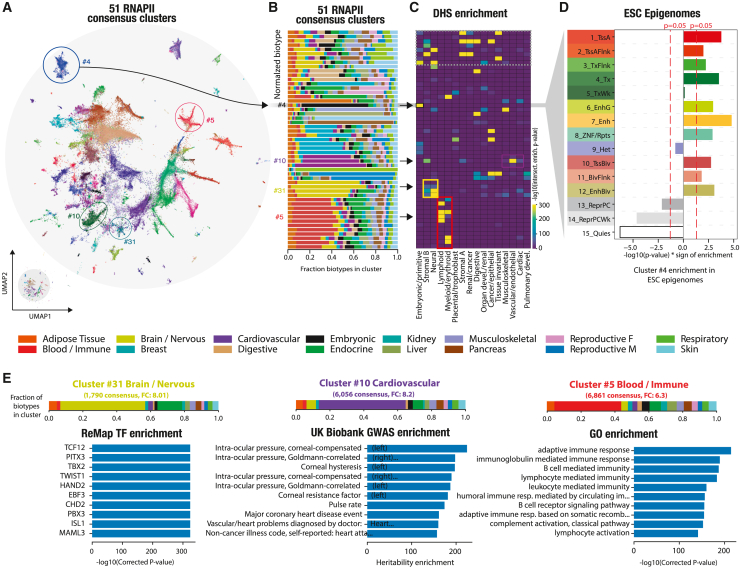
Revealing tissue-specific regulatory signatures (A) Unsupervised graph clustering identifies 51 RNAPII consensus clusters. Four clusters (4, 5, 10, and 31) are highlighted across panels (A)–(C) to illustrate the analysis. (B) The fraction of biotypes within each cluster is shown, indicating tissue-specific or ubiquitous signatures. (C) Enrichment of DNase I hypersensitive site (DHS) biological classification in each cluster. Arrows and colored rectangles highlight correspondence between clusters and DHS categories. (D) Enrichment of ChromHMM epigenetic states of “embryonic stem sell,” sampled at the RNAPII genomic location of cluster 4, against the non-cluster 4 RNAPII consensus. Active states: active TSS transcription states (TssA and TssAFlnk), transcribed promoter and enhancer signatures (TxFlnk), actively transcribed states (Tx and TxWk), enhancer states (Enh and EnhG), zinc-finger protein gene state (ZNF/Rpts). Inactive states: heterochromatin (Het), bivalent regulatory states (TssBiv, BivFlnk, and EnhBiv), repressed Polycomb states (ReprPC and ReprPCWk), and quiescent state (Quies). (E) Top 10 transcription factor enrichments from the ReMap database in cluster 31, top 10 UK Biobank GWAS trait heritability enrichment in cluster 10, and top 10 GO enrichment of nearby genes in cluster 5. All results shown are statistically significant. Each cluster’s biotype distribution is shown as a stacked bar plot.

We next aimed to retrieve and annotate each consensus group to capture its biological identity. Using an unsupervised graph clustering approach, we identified 51 RNAPII consensus clusters (Figure 3A), each harboring its own biotype specificity (Figures 3B and S8; Table S1). To independently validate their biological signatures, we compared the clusters against the biological classification of the human index of DNase I hypersensitive sites^26^ (DHSs) (Figure 3C). The defined RNAPII clusters showed a coherent enrichment with the DHS regulatory vocabulary (Figure S9). For instance, “brain/nervous” RNAPII cluster 31 (light green) was enriched in neural DHSs. To capture the genomic signatures of these groups, we examined the epigenetic state for each RNAPII cluster, particularly focusing on its chromatin state specificity. As an example, we selected RNAPII cluster 4, which exhibited a distinct “embryonic” signature, and analyzed the Roadmap ChromHMM (software for learning and characterizing chromatin states) epigenetic states of embryonic stem cells (Figure 3D). We observed a strong enrichment of “active” epigenetic states, including enhancers, TSSs, and transcribed regions, within the RNAPII embryonic cluster compared with the other RNAPII clusters (Figure S10A; STAR Methods). Conversely, we observed a depletion of “inactive” epigenetic states, such as quiescent or Polycomb-repressed states. This finding suggests that RNAPII occupies intergenic space at key regulatory elements, as demonstrated previously^27^ (Figures 1D and 1E). To explore the tissue specificity of RNAPII clusters, we analyzed enhancer-like histone marks and open chromatin profiles (H3K27ac, ATAC-seq; Table S2). The results revealed that RNAPII cluster-tissue pairs with matching tissues (e.g., heart-cardiovascular) exhibited the strongest activity, while non-matching pairs (e.g., lymphoid-liver) displayed a weaker signal (Figure S10B).

To further confirm the biological identity of defined clusters, we investigated the enrichment of SNP-based trait heritability from a UK Biobank genome-wide association study^24^ (GWAS), transcription factor binding regions (TFBRs) from ReMap,^19^ Gene Ontology (GO) terms, and HOMER^28^ DNA motifs (Figures 3E and S9). “brain/nervous” cluster 31 exhibited enrichment of TFBRs for transcription factors known to be involved in neural development or diseases, such as TCF12, PITX3, and TWIST1. Similarly, at the sequence level, the embryonic RNAPII cluster exhibits enrichments of meaningful transcription factor motifs, specifically OCT4-Sox-NANOG motifs. “Cardiovascular” cluster 10 showed enrichments in multiple heart-related traits, such as intra-corneal pressure, pulse rate, and coronary heart disease. Similarly, blood/immune cluster 5 included an RNAPII consensus located near genes linked to immune response GO terms, consistent with their assigned biotypes. Our study accurately distinguishes intergenic RNAPII occupancy based on its biotype specificity, revealing tissue-specific regulatory signatures across multiple independent genomic resources. These resources range from open chromatin occupancy maps to transcription factor binding, providing comprehensive insights into the regulatory landscape.

### Systematic transcription captured in the intergenic RNAPII atlas

**Figure 4:**
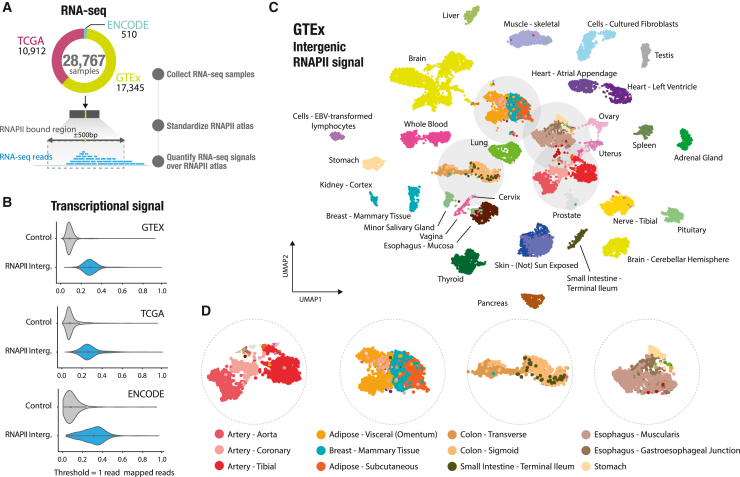
Intergenic transcription on the RNAPII atlas is a powerful indicator for characterizing tissues (A) Number of RNA-seq samples from three expression resources (GTEx, TCGA, and ENCODE) and schematic depicting the standardization of RNAPII consensus to 1-kb RNAPII-bound regions to obtain read counts. (B) Violin plots comparing transcriptional signals at intergenic RNAPII-bound regions versus non-RNAPII random intergenic regions across the three expression resources. (C) Two-dimensional UMAP projection of 17,345 GTEx RNA-seq signals across the intergenic RNAPII atlas, with colors representing 54 tissue types, including 11 distinct brain regions (yellow) and two cell lines (light blue). (D) Magnified view of tissue-specific expression patterns observed in similar tissues, such as different types of artery (e.g., aorta, coronary, and tibial).

We developed the RNAPII atlas as an innovative tool for indirectly identifying intergenic regulatory regions that are active or poised for transcription. To quantify intergenic transcription and gain a better understanding of transcriptional patterns, we utilized the RNAPII atlas to analyze transcriptional signals in three major expression resources. These resources include samples from normal and cancer cell lines: GTEx, TCGA, and the ENCODE consortium. By combining these, we conducted an extensive analysis of intergenic expression across the RNAPII atlas, leveraging data from 28,767 RNA-seq samples (Figure 4A). To quantify intergenic transcription, we first standardized each RNAPII consensus sequence to a 1-kb RNAPII-bound region. We then counted the number of reads that overlapped with these RNAPII -bound regions, generating a count table similar to conventional gene-centric RNA-seq count tables (Figure 4A). Our analysis revealed that the intergenic RNAPII atlas captured approximately 60% of intergenic reads (Figures S11A and S11B). Moreover, these RNAPII-bound intergenic regions captured significantly higher read counts compared with the rest of the intergenic genome. On average, RNAPII -bound regions had 7.13 times more transcriptional signal compared with the remaining intergenic genome (Figures 4B and S11C). By visualizing the spatial distribution of transcriptional signals within RNAPII-bound regions, we discovered two distinct types of transcriptional patterns: one displaying a mono-modal signal with a short peak and the second showcasing a broader peak spanning the entire 1-kb probe (Figure S12). Altogether, the RNAPII atlas is strongly enriched in transcriptional activity, and thus it could serve as a powerful tool for investigating intergenic transcription in normal and cancer tissues.

### Intergenic transcription on the RNAPII atlas is a powerful indicator for characterizing tissues

To determine whether intergenic transcription at RNAPII-bound regions could characterize tissue specificity, we analyzed expression data from 54 non-diseased tissues, comprising a total of 17,345 samples from the GTEx project. Intergenic transcription has been utilized previously as a marker of enhancer activity, as demonstrated in the Functional Annotation of the Mammalian Genome (FANTOM) project,^7^ and across various experimental assays focused on capped and nascent RNAs.^3^^,^^10^^,^^12^^,^^13^ In this study, we developed a pipeline based on single-cell RNA-seq (scRNA-seq) methods, which are commonly employed for analyzing weak signals in datasets with large sample sizes. By considering signals only within RNAPII -bound regions, we were able to extract valuable biological information from read count tables (Figure S13; STAR Methods). We used UMAP to analyze and visualize similarity between the expression levels sampled at RNAPII-bound regions of each GTEx biosample. This analysis revealed a clear distinction between tissues because biosamples originating from the same sampling site are clustered together (Figure 4C). These tissue-specific expression patterns are observed not only between similar tissues, such as artery (aorta, coronary, and tibial) but also between tissues with similar histological features, like adipose tissue (visceral, subcutaneous, and mammary tissue). Additionally, these patterns are observed between tissues located in different body regions, such as the digestive tract (colon and small intestine) (Figure 4D). To test whether intergenic transcription could accurately discriminate the 54 GTEx tissues accurately, we employed a k-nearest neighbor algorithm (KNN classifier) to classify the tissues based on expression of RNAPII-bound regions. This showed that RNAPII-bound regions could predict tissue types with a high level of accuracy, with only a slight decrease in accuracy compared with gene-centric RNA-seq counts processed using the same methods (87.1% against 90.0% balanced accuracy across 54 tissues; Figure S14). Next, we identified overexpressed intergenic RNAPII-bound regions in the GTEx tissues with an average of 4,236 regions per tissue (Figure S15A; STAR Methods). Our analysis revealed a significant association between RNAPII-bound regions with tissue-specific overexpression and tissue-specific GTEx eQTLs (Figures S15B and S15C; STAR Methods), providing evidence that these regions can serve as indicators of transcribed intergenic enhancers. Interestingly, we also detected transcriptional signals at RNAPII-bound regions located downstream of genes (>1 kb), suggesting the presence of transient RNA downstream of the polyadenylation site (Figures S5A and S5B), which is consistent with previous studies.^29^ To further investigate the impact of these downstream signals, we conducted additional analyses excluding RNAPII-bound regions located up to 9 kb downstream of genes. Our findings demonstrate that RNAPII consensus peaks located within the 1- to 9-kb region downstream of genes do not drive classification of GTEx tissues (Figure S16). Furthermore, we show that our approach is applicable to smaller RNA-seq datasets (Figure S17). By comparing the expression levels in three samples of two types of heart tissues from GTEx biosamples, we identified 195 RNAPII-bound regions located near genes related to heart function, despite limited statistical power (Figure S17; STAR Methods). Here, we provide evidence that intergenic transcription detected at RNAPII-bound regions is a strong indicator of tissue specificity and can be used effectively for accurate tissue type prediction. These findings may have implications for understanding tissue-specific gene regulation.

### Meta-analysis reveals tissue- and disease-specific connections between RNAPII occupancy and transcription

**Figure 5:**
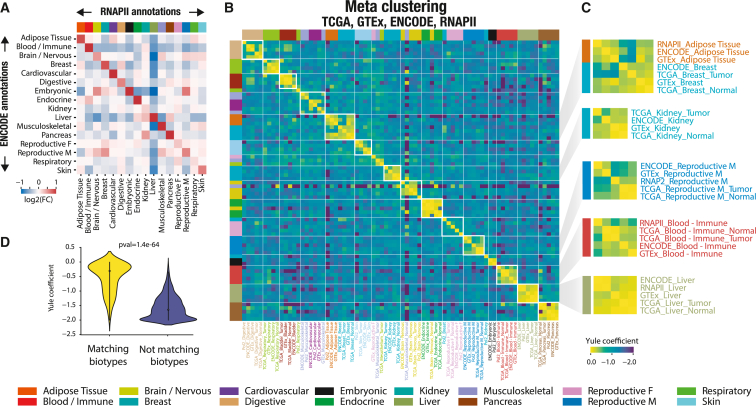
Meta-analysis reveals tissue- and disease-specific connections between RNAPII occupancy and transcription (A) Association between RNAPII occupancy biotype and transcription biotype from ENCODE. The heatmap depicts log2 of ENCODE RNA-seq dataset expression fold change in each biotype (rows) between RNAPII-bound regions with biotype-specific RNAPII ChIP-seq occupancy (columns) against non-specific RNAPII-bound regions. (B) Heatmap showing the association between biotype-specific intergenic RNAPII occupancy and biotype-specific RNAPII overexpression across four resources. A hierarchically clustered heatmap reveals the correct grouping by tissue of origin rather than data source, with each possible biotype-dataset pair represented. Yule distance between a pair of dataset-biotype lists of overexpressed RNAPII markers is indicated. (C) Magnified view revealing meta-clusters of tissue-specific correlation between intergenic RNAPII regions and their transcription in different resources. (D) Distributions of tissue-matching (i.e., RNAPII-liver vs. TCGA-liver) and non-matching (i.e., RNAPII-liver vs. GTEx-heart). Yule distance between two intergenic RNAPII marker sets (p = 1.4e−64) is indicated.

We examined the relationship between biotype-specific RNAPII occupancy and biotype-specific transcription by comparing the observed intergenic signal across all expression datasets, which combined 28,787 RNA-seq samples despite use of different sequencing samples and protocols. We first conducted an analysis to investigate the association between biotype-specific RNAPII occupancy in ChIP-seq and transcription in ENCODE RNA-seq biotypes by comparing biotypes pairwise (Figure 5A). This analysis revealed a significant enrichment of biotype-specific transcription in the ENCODE dataset at RNAPII probes with ChIP-seq occupancy specific to the corresponding biotype, even when considering different samples and protocols. Conversely, non-matching biotype pairs did not exhibit transcriptional signal enrichments. These findings underscore a strong link between RNAPII occupancy and effective transcription as well as the effectiveness of our biosample annotation for comparing varied data sources. Furthermore, we conducted a meta-analysis that integrated every dataset and biotype to obtain a comprehensive and interconnected view of intergenic transcription across nearly 30,000 biosamples from diverse data sources (Figure 5B). In brief, we extracted intergenic RNAPII markers (only considering up-regulated RNAPII -bound regions; STAR Methods) for each possible biotype-dataset pair (i.e., RNAPII-liver, GTEx-heart, and ENCODE-liver) and quantified pairwise similarity between marker lists for every biotype-dataset combination, assuming that a marker list is characteristic of a specific biotype. We then applied hierarchical clustering to generate a meta-clustering that revealed similarities between tissues across all resources (Figure 5B; STAR Methods). This meta-analysis highlighted that the association between intergenic RNAPII occupancy and intergenic transcription is biotype specific, consistently observed across biotypes and independent of dataset origins or protocols used. Our approach effectively grouped similar biotypes together, independent of the data source (Figure 5B). For instance, “adipose tissue” and “Breast” tissues clustered together across resources, reflecting the presence of adipose cells in breast tissue (Figure 5C). Moreover, identical biotypes exhibited much greater similarity in markers across data sources than non-identical biotypes (Figure 5D). To ensure robustness, we extracted markers that were supported by at least half of the data sources for each biotype. These markers demonstrated a strong enrichment of heritability in biotype-related traits, confirming their biological relevance (Figure S18). For instance, markers associated with the “reproductive female” biotype showed a strong correlation with heritability of the “birth weight of first child” trait, while markers associated with the “liver” exhibited enrichment in heritability for “high cholesterol.” In summary, our meta-analysis revealed a tissue-specific correlation between intergenic transcription and RNAPII occupancy, which carries biological significance. Furthermore, we observed a remarkable consistency across diverse data sources and protocols.

### Cancer type and subtype classification by intergenic transcription at RNAPII binding sites

**Figure 6:**
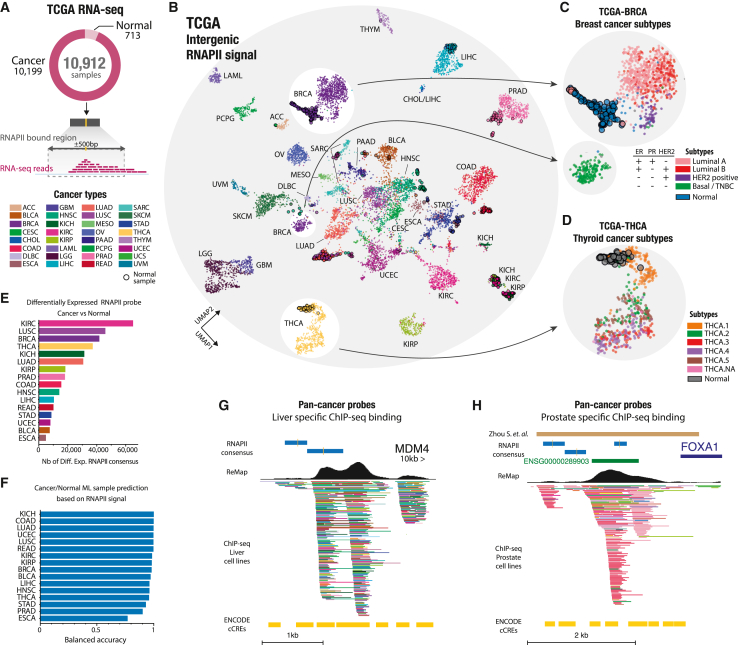
Cancer type and subtype classification by intergenic transcription at RNAPII binding sites (A) A total of 10,912 TCGA RNA-seq samples were leveraged to capture intergenic signals at standardized RNAPII 1-kb bound regions. (B) A two-dimensional UMAP of 10,912 TCGA patients based on intergenic RNAPII transcriptional signals. Each dot represents a TCGA cancer patient or normal sample, with the colors representing the cancer type. White circles highlight breast cancer (BRCA) and thyroid carcinoma (THCA) samples. (C and D) Magnified projections of distinct BRCA and THCA patients (dots) colored by subtype categories based on intergenic transcriptional signals. Normal samples have larger solid black outlines. (E) Number of tumor-specific intergenic RNAPII-bound regions differentially expressed in tumors compared with normal samples. (F) Machine learning classification performance (balanced accuracy) between normal and tumor samples for each cancer type. (G) Genomic view of a pan-cancer intergenic RNAPII-bound region differentially expressed in seven or more cancers. Two pan-cancer markers are located on enhancers (enhancer distal, cCREs) near the MDM4 gene with ChIP-seq bindings. (H) The brown bar represents a published *cis*-regulatory element of FOXA1 harboring somatic variants in primary prostate tumors.^38^ ChIP-seq ReMap tracks are filtered to show transcription factor (TF) binding specifically in liver or prostate cell lines.

We have shown that intergenic transcription can reliably differentiate between various tissues and biological conditions. Expanding on this understanding, we explored direct applications of our RNAPII atlas and its potential implications in human cancers. We analyzed expression data from 32 cancer types, encompassing 10,912 RNA-seq samples obtained from the TCGA cohort, to identify clinically relevant intergenic transcription patterns and potential therapeutic targets (Figure 6A). Using UMAP, we analyzed and visualized the similarity in expression profiles among biosamples, revealing an initial separation between cancer types and subsequently between normal or tumoral tissue states, suggesting that certain RNAPII-bound regions are differentially expressed in these contexts (Figure 6B). For example, in the case of brain cancers, lower-grade glioma (LGG) and Glioblastoma multiforme (GBM) display close clustering, whereas kidney tumor samples (kidney renal clear cell carcinoma [KIRC], kidney chromophobe carcinoma [KICH], and KIRP) exhibit distinct expression profiles despite the similarity observed in normal kidney samples. Interestingly, breast cancer (BRCA) samples form two distinct clusters based on expression of intergenic RNAPII-bound regions. These clusters correspond to distinct BRCA subtypes, with the basal-like subtype (triple-negative BRCA [TNBC]) being the most distinct and the luminal A, luminal B, and HER2-positive subtypes forming a separate, larger group (Figure 6C). We identified intergenic transcriptional markers specific to the basal-like/TNBC subtype, which are associated with 10 dual-specificity phosphatase genes (e.g., DUSP1, DUSP5, and DUSP7), involved in mitogen-activated protein kinase (MAPK) phosphatase activity. MAPK cascades play a central role in cell proliferation and apoptosis, and DUSP1 may contribute to development of chemoresistance in TNBC.^30^^,^^31^ TNBC accounts for approximately 15%–20% of all BRCA cases, is most prevalent in women under 40,^32^ and presents aggressive behavior.^33^ Similar to BRCA, intergenic transcription in thyroid carcinomas (THCA) facilitated the identification of different subtypes of THCAs (Figure 6D). By using a heatmap representation of the differentially expressed RNAPII-bound regions in KICH samples, we observed distinct clusters of up-regulated and down-regulated RNAPII -bound regions. These clusters indicate potential tumor subtypes with unique intergenic expression patterns (Figure S19A). Identification of subtype-specific intergenic transcription sheds light on cancer biology by revealing active regulatory elements and potentially actionable nearby genes with clinical significance.

### Identification of per-cancer and pan-cancer intergenic transcriptional markers

We identified tumor-specific RNAPII-bound regions differentially expressed in tumors compared with normal tissues for 16 cancer types, ranging from 65,050 regions for KIRC to 6,458 regions for ESCA (esophageal carcinoma) (Figure 6E). These numbers align with previously identified active enhancers in TCGA cancers.^34^ The predictive power of these regions was confirmed as we accurately separated tumors from normal tissues in most cancers using a machine learning classifier (Figure 6F; STAR Methods). To uncover pan-cancer intergenic transcriptional markers that could contribute to tumorigenesis across multiple cancer types, we identified RNAPII-bound regions differentially expressed in a substantial number of cancers (7 or more of 16; Figure S19B; STAR Methods). We observed a large number of RNAPII-bound regions that did not appear to be differentially expressed in any specific type of cancer. However, on the other end of the spectrum, we observed a significant number of RNAPII-bound regions that exhibited differential expression in a greater number of cancers than expected. Specifically, we found 10,940 RNAPII-bound regions to be differentially expressed in more than seven cancers, with some expressed in each of the 16 cancers that had corresponding normal tissue samples available. Within this set of 10,940 pan-cancer differentially expressed RNAPII-bound regions, we identified previously known regions implicated in cancer as well as new loci (Figures 6G and 6H). For example, we identified two pan-cancer differentially expressed RNAPII-bound regions on enhancers located 10 kb upstream of the MDM4 gene (Figure 6G). This protein is involved in repression of the tumor suppressor TP53 and represents a potential therapeutic target in liver cancer^35^ and lymphomas^36^ and overall in anticancer therapy.^37^ Additionally, we highlighted a group of pan-cancer RNAPII-bound regions that overlapped a known, frequently mutated^38^ FOXA1 enhancer region involved in proliferation of prostate cancer cells (Figure 6H). This region has been identified as one of six *cis*-regulatory elements in the FOXA1 regulatory plexus harboring somatic single-nucleotide variants in primary prostate tumors.^38^ FOXA1 acts as a pioneer factor in prostate cancer and governs expression of cell cycle regulatory genes in prostate cancer. Overall, these 10,940 regions appear to be located near cancer hallmark genes (Figure S19C). Our analysis revealed differentially expressed intergenic markers in tumors or tumors subtypes compared with normal tissues, which may directly or indirectly contribute to tumorigenesis. By identifying potential intergenic transcriptional markers, our findings could pave the way for novel therapeutic strategies targeting clinically actionable genes.

### Intergenic transcriptional markers showing clinical relevance in cancer

**Figure 7:**
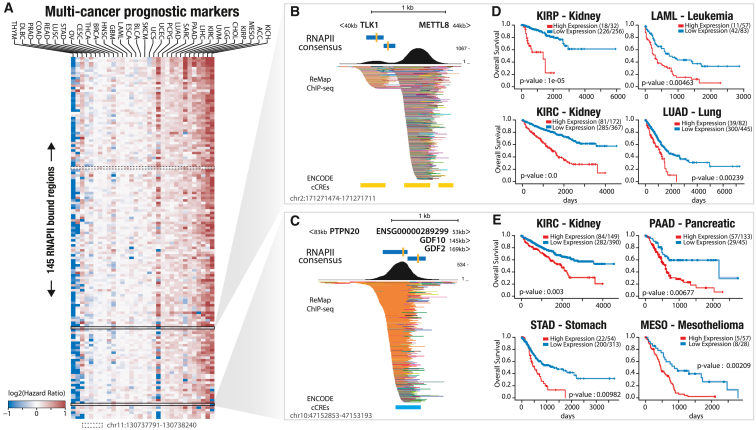
Intergenic transcriptional markers showing clinical relevance in cancer (A) Heatmap of 145 transcribed RNAPII-bound regions identified as prognostic markers in multiple cancers. A color scale depicts log2(hazard ratios) of strong expression associated with a good (blue) or bad (red) prognosis. Black rectangles highlight two intergenic prognostic markers (RNAPII-bound regions in B and C). A dashed-line rectangle highlights a prognostic marker shown in the supplementary. (B) Genomic landscape of identified multi-cancer prognostic markers (blue bars) at chr2:171,271,474–171,271,711 located 40 kb downstream of the TLK1 gene and 44 kb upstream of the METTL8 gene. Yellow bars indicate candidate *cis*-regulatory elements (cCREs, enhancer distal) and ChIP-seq binding from ReMap. (C) Genomic view of the multicancer prognostic markers (chr2:171,271,474–171,271,711) located 83 kb downstream of the PTPN20 gene and 145/196 kb downstream of the GDF10 and GDF2 genes as well as 53 kb downstream of a, lncRNA gene, ENSG00000289299. A light blue bar indicates a cCRE CTCF region. (D) Kaplan-Meier survival analysis of kidney cancer (papillary and clear), leukemia, and lung cancer patients with high (red) and low (blue) expression from the intergenic RNAPII-bound region in (B). (E) Kaplan-Meier survival analysis of kidney, pancreatic, stomach, and mesothelioma TCGA cancer patients with high (red) and low (blue) expression from the intergenic RNAPII-bound region in (C).

To examine the clinical relevance of intergenic transcriptional markers, we investigated the association between expression of RNAPII-bound regions and overall survival per cancer and pan cancer using a Cox proportional hazard model (per- and pan-cancer marker lists and count tables are available at Zenodo^18^). At the per-cancer level, our results showed a smaller number of RNAPII-bound regions associated with overall survival compared with previous analyses of differentially expressed RNAPII regions. The largest number of associated regions was observed in LGG (n = 18,380), with an average of 2,002 regions per cancer (Figure S20A). At the pan-cancer level, we identified a set of 145 RNAPII-bound regions associated with overall survival in five or more cancer types. Most of these regions showed a positive association between overexpression and poor survival (hazard ratio > 1; Figure 7A). The 145 RNAPII-bound regions identified were found to be in close proximity to genes involved in the cell cycle, DNA metabolism and repair, and muscle development as well as hallmark genes of genome instability and mutation (Figures S20B and S20C). Perturbation and acceleration of the cell cycle are hallmarks of cancer and play a role in tumor progression and prognosis. As examples, we highlight two RNAPII-bound regions associated with overall survival (OS) and located near known cancer-associated genes and candidate regulatory elements (Figures 7B, 7C, and S21).

The first RNAPII-bound region is located between the genes TLK1 and METTL8 at 40 kb and 44 kb, respectively (Figure 7B). TLK1 has been linked to poor patient outcomes in multiple cancer types, including GBM^39^ and prostate cancer metastasis,^40^^,^^41^ and it is involved in DNA replication and chromatin assembly.^39^ METTL8 has been identified as a potential biomarker in hepatocellular carcinoma,^42^ and high levels have been associated with improved patient survival in pancreatic cancer.^42^ We observed that high expression of the pan-cancer RNAPII-bound region depicted in Figure 7B is strongly linked to survival in leukemia and kidney (KIRP and KIRC) and lung cancers (Figure 7D).

The second region is located between three genes: downstream of a protein tyrosine phosphatase non-receptor (PTPN20) at 83 kb and upstream of two growth differentiation factors, GDF10 and GDF2, at 145 kb and 196 kb, respectively (Figure 7C). GDF10 and GDF2 belong to the transforming growth factor β (TGF-β) superfamily and are considered tumor suppressors^43^ in certain cancers. Studies have demonstrated that GDF10 expression is an independent prognostic factor for OS of patients with oral squamous cell carcinoma.^44^ Additionally, GDF10 inhibits cell proliferation and epithelial-mesenchymal transition in nasopharyngeal carcinoma.^45^ On the other hand, GDF2 exhibits pleiotropic effects in tumorigenesis, promoting ovarian cancer cell growth^46^ while suppressing breast tumorigenesis^47^ and increasing hepatocellular carcinoma cell growth.^48^ It also plays a role in suppressing cell death in ovarian and breast epithelia.^49^ Furthermore, numerous protein tyrosine phosphatases have been shown to regulate essential cellular processes, with several mutations associated with human diseases.^50^ This pan-cancer RNAPII-bound region demonstrates not only a previously established correlation with mesothelioma cancers but also a new correlation between its expression and survival rates in kidney, pancreatic, and stomach cancers (Figure 7E). Taken together, these analyses suggest that these transcribed RNAPII regions, which are mostly unreferenced and undetected, may have clinically relevant roles in cancer and could serve as potential markers for OS. Additional studies are needed to fully understand the potential clinical implications of these observations.

## Discussion

We constructed an atlas of intergenic transcription at RNAPII binding sites to connect genomic, transcriptomic, and clinical data across normal tissues and cancer samples. Our approach utilizes a normalized vocabulary for cell lines and tissue types and integrates a compendium of 906 publicly available RNAPII ChIP-seq profiles, enabling comprehensive exploration of intergenic transcription across 28,000 expression samples. The atlas provides an efficient means to investigate tissue specificity and the activity of core regulatory elements in various tissues. Our meta-clustering approach reveals that transcription of intergenic regions is shared among similar tissues and across multiple independent resources. We identified per-cancer and pan-cancer intergenic transcriptional markers associated with known cancer genes and prognostic intergenic markers that predict overall patient survival. Additionally, we discovered that intergenic transcriptional markers can effectively discriminate between subtypes of breast and thyroid cancers.

Our mapping of intergenic transcription stands out from prior efforts to characterize enhancer activities because we directly target the RNAPII transcriptional machinery. Traditionally, studies have relied on single resources, such as histone signatures from ENCODE or Cap Analysis of Gene Expression (CAGE) transcripts from FANTOM, to identify non-coding elements. However, our study demonstrates the effectiveness of robust data integration using diverse public RNAPII ChIP-seq datasets, providing a coherent method to characterize intergenic transcriptional activity in normal and cancer tissues. To detect non-coding transcription, previous investigations^3^^,^^8^^,^^27^^,^^51^ have utilized techniques such as GRO-seq or its derivatives to capture nascent transcripts. However, these techniques have limitations in terms of coverage and representation of normal tissues and cancer types. In our study, we address these limitations by integrating GTEx, TCGA, and ENCODE RNA-seq data, offering new insights into intergenic activity across cell lines, normal tissues, and cancer types.

The intergenic RNAPII consensus peaks observed in our study exhibit characteristics suggestive of enhancers, potential TSSs, or potential transcription termination sites (TTSs) that have yet to be described. The majority of identified intergenic RNAPII consensus peaks were classified as regulatory regions, with 65.9% displaying an enhancer signature and 17.4% located downstream of genes. These findings align with the current understanding of the non-coding genome, where enhancers and other regulatory elements are known to recruit RNAPII. In this study, we annotate all 181,547 intergenic RNAPII consensus with genomic characteristics and biotype signatures. Our large-scale integration approach enabled comprehensive exploration of intergenic transcription in normal tissues and cancer types.

Using signals from cancer transcriptomes (TCGA), we identified differentially expressed RNAPII intergenic regions and molecular subtypes of breast and thyroid cancers. For example, in TNBC, we showed that certain differentially expressed RNAPII regions are located near DUSP genes involved in the MAPK signaling pathway. This pathway plays a crucial role in regulating cell proliferation and apoptosis, and DUSP1 in particular may contribute to chemoresistance in TNBC.^30^^,^^31^ While we demonstrated that RNAPII consensus targets intergenic enhancer elements or proximal enhancers upstream of genes, we also observed RNAPII consensus located downstream of gene TTSs. Future investigations may help identify new sites of transcription termination across our biotype panel.

Transcription of non-coding regions is a fundamental characteristic captured by our RNAPII intergenic map across cell lines, normal tissues, and cancer samples. This significantly expands the analysis horizon beyond gene-centric annotations. Our integration framework symbolizes a transition from exploratory studies centered around uncovering new regulatory elements to a map-focused phase that prioritizes identification of active transcribed elements within specific biological contexts. The significance of our study lies in its ability to enhance our understanding of the activity of non-coding regions in cancer biology and disease development, potentially guiding therapeutic approaches and ultimately improving patient outcomes.

### Limitations of the study

Despite the valuable insights gained from our study, there are some limitations that should be considered. One limitation is the reliance on publicly available RNAPII ChIP-seq datasets, which may introduce potential biases and variations in experimental conditions. Additionally, the analysis focused on RNAPII-bound regions and their transcriptional activity, but the nature of the produced transcripts or other factors, such as chromatin conformation, were not directly addressed. Moreover, the use of transcriptomic data from public databases may not fully represent all tissue types and cancer subtypes, potentially limiting the generalizability of our findings. While our meta-clustering approach allowed us to integrate diverse datasets, some tissues or cell types may still have limited representation, affecting the accuracy of tissue-specific classifications. Future experiments with larger and more diverse datasets would be necessary to validate and expand the conclusions drawn from this study. Nonetheless, our atlas of intergenic transcription at RNAPII binding sites offers a valuable resource for investigating tissue-specific regulatory elements and holds promise for advancing our understanding of non-coding transcription in normal and cancer tissues.

## STAR★Methods

### Key resources table

**Table:** 

REAGENT or RESOURCE	SOURCE	IDENTIFIER
**Deposited data**		

UK Biobank	UK Biobank^67^	https://www.ukbiobank.ac.uk/
UK Biobank GWAS summary statistics	UK Biobank^67^	http://www.nealelab.is/uk-biobank
UK Biobank GWAS summary statistics	UK Biobank^67^^,^^68^	https://pan.ukbb.broadinstitute.org
RNAPII ChIP-seq summary	This paper	Table S1
Tissue-wide RNA-seq expression	GTEx project^15^	https://gtexportal.org/home/datasets
Cell lines RNA-seq expression	ENCODE project^1^^,^^20^	https://www.encodeproject.org/
Cancer samples RNA-seq expression	TCGA project^16^	http://gdc.cancer.gov
Tissue specific eQTL v8	GTEx project^15^	https://gtexportal.org/home/datasets
Human genome annotation v38	GENCODE project^56^	https://www.gencodegenes.org/
Human ChIP-seq peaks	ReMap project^19^	http://remap.univ-amu.fr/
ENCODE backlisted regions	Amemiya et al.^17^	https://github.com/Boyle-Lab/Blacklist/
Human candidate Cis Regulatory Elements	ENCODE project^1^^,^^20^	https://www.encodeproject.org/
DNase I hypersensitive	Meuleman et al.^26^	https://doi.org/10.1038/s41586-020-2559-3
Long non-coding RNA encyclopedia v5	LNCipedia^23^	https://lncipedia.org/
Transcribed Enhancers CAGE eRNAs	FANTOM5 project^21^^,^^22^	https://fantom.gsc.riken.jp/
ENCODE STARR-seq	ENCODE project^1^^,^^20^	https://www.encodeproject.org/
Repeat elements	UCSC Genome Browser^57^	https://genome.ucsc.edu/
H3K27Ac ChIP-seq experiments	ENCODE project^1^^,^^20^	Accession IDs are in Table S2
ATAC-seq experiments	ENCODE project^1^^,^^20^	Accession IDs are in Table S2
Epigenome states (Core 15-states model)	ROADMAP project^63^	https://egg2.wustl.edu/roadmap/web_portal/chr_state_learning.html
Cancer Hallmarks Genes database	CHG database	http://www.bio-bigdata.com/CHG/

**Software and algorithms**		

Bowtie2 (2.4.2)	Langmead and Salzberg^53^	http://bowtie-bio.sourceforge.net/bowtie2/index.shtml
Samtools (1.11)	Li et al.^74^	http://samtools.sourceforge.net/
DESeq2 (1.40.2)	Love et al.^73^	https://bioconductor.org/packages/release/bioc/html/DESeq2.html
ReMap ChIP-seq pipeline (2022)	Hammal et al.^19^	https://github.com/benoitballester/
Trim Galore (0.6.5)	Babraham Bioinformatics	https://github.com/FelixKrueger/TrimGalore
MACS2 (v2.1.2)	Zhang et al.^55^	https://github.com/macs3-project/MACS
Python (3.6.12)	Python Core Team	https://www.python.org/
Python PyRanges library (0.0.129)	Stovner and Saestrom^58^	https://pypi.org/project/pyranges/
Python fastcluster library (1.2.6)	Müllner, D.^60^	https://pypi.org/project/fastcluster/
Python Pynndescent library (0.5.10)	Dong et al.^61^	https://pypi.org/project/pynndescent/
Python Statsmodels library (0.14.0)	Python Package Index (PyPI)	https://pypi.org/project/statsmodels/
Python Lifelines library (0.27.7)	Python Package Index (PyPI)	https://pypi.org/project/lifelines/
Python kaplanmeier library (0.1.9)	Python Package Index (PyPI)	https://pypi.org/project/kaplanmeier/
Pandas	https://pandas.pydata.org	RRID:SCR_018214
Numpy	http://www.numpy.org	RRID:SCR_008633
Scipy	https://www.scipy.org	RRID:SCR_008058
scikit-learn	http://scikit-learn.org	RRID:SCR_002577
R (4.0.5)	R Core Team	https://www.R-project.org
R maxstat library (0.7–25)	CRAN project	https://cran.r-project.org/package = maxstat
deepTools (3.5.2)	Ramirez et al.^64^	https://github.com/deeptools/deepTools
GREAT (v4.0.4)	Mc Lean et al.^65^	http://great.stanford.edu/public/html/
HOMER (v4.11)	Heinz et al.^28^	http://homer.ucsd.edu/homer/
Stratified LD-Score Regression	Finucane et al.^66^	https://doi.org/10.1038/ng.3404
featureCounts	Liao et al.^69^	https://doi.org/10.1093/bioinformatics/btt656
SCTransform	Choudhary et al.^71^	https://doi.org/10.1186/s13059-021-02584-9

**Other**		

Code for main figures and analysis	This paper; Github	https://github.com/benoitballester/Pol2Atlas
Intergenic RNAPII Atlas: input data	This paper; Zenodo	https://zenodo.org/record/7785393
Intergenic RNAPII Atlas: output data	This paper; Zenodo	https://zenodo.org/record/8091826
Intergenic RNAPII Atlas: annotated consensus	This paper	Data S1

### Resource availability

#### Lead contact

Further information and requests for resources should be directed to and will be fulfilled by the lead contact, Benoit Ballester (benoit.ballester@inserm.fr).

#### Materials availability

This study did not generate new unique reagents.

### Method details

#### RNAPII ChIP-seq data processing

We recovered from NCBI-GEO all existing RNA Polymerase II (RNAPII) ChIP-seq experiments targeting the POLR2A subunit (n = 1,135) in human, following the ReMap procedures and pipeline.^19^ Briefly, we manually annotated and standardised the cell line and tissue of origin names (Table S1). Every experiment was downloaded and processed uniformly starting from the fastq files, to quality checks, up to the peak calling stage using the ReMap pipeline. In more detail, ChIP-seq experiments were retrieved from the NCBI Gene Expression Omnibus (GEO) and ENCODE databases. For GEO, the query ‘Genome binding/occupancy profiling by high-throughput sequencing’ AND ‘homo sapiens’[organism] AND NOT ‘ENCODE’[project]’ was used to return a list of all potential studies. The selected experiments metadata are then manually curated and annotated with official nomenclatures for target names and biotypes. For incomplete metadata, the materials and methods of associated and published papers are often examined to complete the curation. We used the BRENDA Tissue Ontologies for cell lines at the EBI Ontology Lookup Service (www.ebi.ac.uk/ols/ontologies/bto) as well as the Cellosaurus database to homogenize cell and tissue names (e.g., MCF-7 not MCF7, Hep-G2, not HepG2, Hepg2 etc.). We define a dataset as a DNA-binding experiment in a given GEO/ENCODE series (e.g., GSE37345), for a given RNA Polymerase II subunit (e.g., POLR2A), and in a particular biotype (e.g., LNCaP, MCF-7) in a given biological condition. Datasets are labeled with the concatenation of these information (e.g., GSE37345.POLR2A.LNCAP_45min-DMSO). All data were re-analysed starting from raw FASTQ files. Both GEO and ENCODE datasets were manually curated, processed and analyzed in the same way. Bowtie 2^53^ (version 2.2.9) with options -end-to-end -sensitive was used to align all reads on the human genome GRCh38/hg38 assembly. Trim Galore (https://github.com/FelixKrueger/TrimGalore) was used to remove adapters, trimming reads up to 30 bp. Trim Galore is a wrapper around Cutadapt and FastQC to consistently apply adapter and quality trimming to FastQ files. With samtools rmdup polymerase chain reaction duplicates were removed from the alignments. Following the ENCODE ChIP-seq guidelines^54^ we used the MACS2^55^ peak-calling tool (version 2.1.1.2) to identify the RNAPII-bound regions. For all the datasets, the corresponding bed file is available for download. In order to study only the intergenic part of the genome, we filtered out peaks overlapping GENCODE^56^ v38 transcripts ±1kb. We also excluded ENCODE blacklisted regions.^17^ We retained peaks with a MACS2 q-value under 10^−5^, and removed uninformative datasets with less than 100 intergenic peaks. In the end, we conserved 906 out of 1,135 datasets after all Quality Checks (Figures S1B and S1D). Finally we investigated the distribution of antibody usage across the 906 datasets (Figure S1A). The Table S1 includes standardised antibody information, which was manually curated from ENCODE, GEO or the associated paper methods.

#### High level biosample annotation

Due to the very large biological diversity of the experiments, it is necessary to have a high level annotation to make the interpretation of the results easier, as well as comparing results between datasets. We annotated samples according to their tissue of origin, with the simplified GTEx tissue (30 tissues) annotation as a baseline, to which we added additional tissues: bone, eye, embryo and trachea. To make some results more interpretable, we grouped similar tissues (e.g., various brain tissues into ‘Brain’) obtaining an annotation with 18 categories (Table S3). A full sample-annotation table is available in Table S1.

#### Construction of the intergenic RNAPII atlas

A naive approach to delineate groups of RNAPII peaks corresponding to a similar biological signal across experiments would be to merge overlapping peaks. However, when the number of experiments is large, the entire genome becomes covered with peaks which makes this approach impractical. To create consensus RNAPII peaks, we first computed the density function of the peak summits (the single base pair genomic location with the maximum signal of the peak) across each chromosome. Due to the inherent inaccuracy on the summit position of these sequencing techniques and the undersampling, this estimate is extremely noisy. To reduce the amount of noise, we applied a Gaussian filter to this density function across the genome (Figure 1A). Consensus peaks were defined at each local minima of the smoothed density function. A peak belongs to a consensus if its peak summit falls in between the identified flanking local minimas. The boundaries of the defined consensus peak were reduced to the ones of the farthests peaks. By default 1/8th of the average peak size was used as the standard deviation of the Gaussian kernel, and to be valid each consensus was required to contain at least 2 peaks from different experiments. Consensus peaks centroid were defined as the mean position of the peak summits. The middle of the peak was used, if a summit coordinate was not available. A binary data matrix was generated to summarise all datasets. For each consensus peak, this matrix stores if a biosample has a RNAPII peak that belongs to it, similar to the DNAse 1 binary matrix from ENCODE.^26^ A schematic of the whole approach is available in Figure S1. Identified and annotated RNAPII consensus are available in Data S1.

#### Comparison with reference databases from other large-scale efforts

The RNAPII atlas was intersected against GENCODE^56^ v38, LNCipedia^23^ v5, FANTOM5,^21^^,^^22^ ReMap^25^ 2022, ENCODE cCREs,^20^ ENCODE STARR-seq and Repeat elements downloaded from UCSC^57^ (hg38). Intersections are computed using the centroid of RNAPII consensus (1bp) against the whole genomic features. The PyRanges python library^58^ was used to compute intersections between genomic features. We computed overlap enrichments for the whole dataset using a binomial test where: n, the number of trials, is the number of RNAPII consensus; p, the probability of intersection, is the base pair coverage of the feature of interest divided by the coverage of the intergenic regions (+-1kb from genes, excluding ENCODE blacklisted regions); k, the observed number of successes, is the number of RNAPII consensus intersecting the feature of interest; The fold change is computed as $\frac{k/n}{p}$. We computed overlap enrichments for subsets of the whole RNAPII atlas using a hypergeometric test, which removes the RNAPII-specific intersection bias: where N, the population size, is the number of RNAPII consensus; K, the number of successes in the population, is the number of RNAPII consensus intersecting the feature of interest; n, the number of draws, is the number of RNAPII consensus of the subset of interest; k, the number of observed successes, is the number of RNAPII consensus of the subset of interest intersecting the feature of interest.

#### Annotation of RNAPII consensus

We performed functional annotation of the 181,547 RNAPII consensus using a simplified approach, where each RNAPII consensus can overlap multiple categories such as Promoter-like, LNC-body, Enhancer-like, Regulatory-like, Gene-tail, Unannotated (Figure S4A). In the following analyses, genomic intersections are performed at the RNAPII consensus centroid against the whole genomic feature. Promoter-like: RNAPII consensus were annotated as promoter-like if they met any of the following criteria: presence of cCREs PLS (Promoter-like Sequence) or cCREs H3K4Me3 (histone H3 lysine 4 trimethylation) or F5 TSS (FANTOM5 Transcription Start Site) or if they overlapped with a LNCipedia promoter, a comprehensive database of long non-coding RNA transcripts. Here, LNCipedia promoters are defined as ±1kb regions around their promoter. LNC-body (potential LNC RNA transcripts): RNAPII consensus were annotated as LNCipedia transcripts if they matched any of the transcripts present in the LNCipedia database, excluding those already annotated as Promoter-like. Here LNCipedia transcripts are extended by 1kb. Enhancer-like: RNAPII consensus were classified as Enhancer-like if they fulfilled the following criteria: presence of cCREs ELS (Enhancer-like Sequence) or F5 Enhancer (FANTOM5 Enhancers) or if they showed overlap with enhancer regions identified by ENCODE STARR-seq. RNAPII consensus that were already annotated as Promoter-like were excluded from this category. Regulatory-like: RNAPII consensus were labeled as unannotated regulatory if they met the criteria of being present in ReMap CRM or ENCODE DNase (DNase I hypersensitivity sites) datasets. Additionally, they were excluded if they were already classified as Promoter-like, Enhancer-like, or LNC-like. Gene-tail: RNAPII consensus were labeled as gene-tail if they were located +1kb to +9kb downstream of a GENCODE gene. Unannotated: RNAPII consensus that did not fulfill any of the aforementioned criteria were considered unannotated. By applying these specific criteria, we were able to assign functional annotations to 90.9% RNAPII consensus, enabling us to gain insights into their putative regulatory roles and characterising their potential functional significance within the context of our study. In addition, we compared the RNAPII consensus to reference databases through a more detailed and non-exclusive analysis (Figure S6). Identified and annotated RNAPII consensus are available in Data S1.

#### RNAPII atlas visualisation and clustering

To visualise the similarity between datasets, we applied UMAP with the Yule similarity, with 30 neighbors and the minimum distance set to 0.5. To visualise the similarity between RNAPII consensus, we use the Sorensen-Dice similarity, 30 neighbors and the minimum distance is set to 0. Other parameters were left to default. For the consensus peaks UMAP, to highlight consensus peaks specific to a biotype annotation, each consensus peak was colored by its most frequent biotype annotation. To do so, we compute the sum of the number of peaks per dataset of each annotation at each consensus ($s_{i,j})$, which is then normalised by the total number of peaks for each annotation ($n_{i,j}$): $s_{i,j}=\sum_{k=1}^{N}M_{k\;\epsilon \;a_{i},j}$ and $n_{i,j}=\frac{s_{i,j}}{\sum s_{i,*}}$, where $a_{i}$ is a set storing the index of each datasets belonging to annotation i, M is the dataset binary matrix, N the number of experiments, and j the consensus index. This prevents over-represented annotations or annotations with some datasets with a large number of peaks to annotate most of the consensus peaks. We chose as representative for consensus j the annotation i for which $n_{i,j}$ is the largest.

Finally, to identify consensus peaks that are not condition specific, each one is linearly grayed according to its Gini-Simpson index: $\lambda_{j}=1-\sum_{i=1}^{R}p_{i,j}^{2}$, where R is the number of annotations, and $p_{i,j}=\frac{n_{i,j}}{\sum n_{*,j}}$. The Gini-Simpson index is a measure of diversity: in this study, it tends toward one if the annotations are equidistributed, and is equal to zero if the consensus only has peaks belonging to datasets with the same annotation.

A three steps Hierarchical Clustering (HC) approach was used to order datasets and RNAPII consensus peaks. First, we performed a UMAP dimensionality reduction to 10 dimensions, and used the same metric as the 2D UMAP transform. This step allows the use of any metric, as UMAP optimises to a lower dimensional space using the euclidean distance, which is used by k-means and Ward HC, and also improves k-means and HC quality. Second, we reduced the effective number of points using k-means clustering and grouped very similar points into 50,000 clusters (step performed only when >50,000 points). This approach is documented and allows to scale Ward HC to very large datasets.^59^ Third, we performed Ward HC on the k-means clusters centroids using the fastcluster library.^60^ The bottom part of the heatmap displays each $p_{i,j}$ as defined in the previous section as a stacked barplot.

A Shared Nearest Neighbor (SNN) Graph Clustering approach was used to identify clusters of RNAPII consensus peaks. This approach is common in single-cell RNA sequencing (scRNA-seq) analyses to identify clusters of cells without *a priori* on the number of clusters. To scale to a large number of points to cluster, we used an Approximate Nearest Neighbor (ANN) method to build the NN graph (python library pynndescent^61^). This approach avoids the quadratic time complexity of building exact nearest neighbors, can use any metric and runs in an almost linear time complexity. The Sorensen-Dice coefficients were used to measure distances between points. In the SNN graph, vertices are weighted by the number of shared nearest neighbors between the two nodes. To identify communities in the SNN graph, we used the Leiden graph clustering algorithm implemented in the python leidenalg^62^ library.

#### Extending the integrative approach to H3K27ac ChIP-seq

We collected all H3K27ac ChIP-seq experiments from ENCODE, retrieved processed files in bed narrowPeak format, mapped for hg38, and without audit error appearing on the sample metadata (n = 890 samples). Each sample was annotated with the same biotype methodology as our RNAPII consensus. The same integrative approach and settings as the RNAPII atlas presented above were run on this dataset.

#### Epigenetic enrichments

We downloaded every 15 states epigenome available from ROADMAP^63^ (hg38). We intersected (consensus centroid only) each consensus peak with each epigenome to get the epigenetic state of each consensus in each epigenome. We computed the proportion of epigenetic states for the subsets/clusters of RNAPII consensus of interest for each epigenome (i.e., the sum of the epigenetic states proportions is equal to one in an epigenome). We used the “GROUP” column of the epigenome metadata to annotate and group epigenomes. We used a paired t-test to statistically assess the difference in proportions between subsets of RNAPII consensus across epigenomes. We downloaded H3K27ac ChIP-seq and ATAC-seq processed bam files from ENCODE for Heart, Liver and T cells samples (Table S2). We used deepTools^64^ to compute the mean profiles at each RNAPII consensus of the studied clusters (+-5kb from centroid).

#### Gene Ontology enrichments of nearby genes

To assign consensus peaks to genes, we used a similar heuristic as GREAT^65^ at default settings: a basal domain of 5kb upstream and 1kb downstream, extended in both directions up to 1Mb or the nearest basal domain (whichever is the closest). For each gene we obtained the number of consensus peaks in its regulatory region, for all the consensus peaks (n) and its subset of interest (k). To compute Gene Set enrichments, we used a Negative Binomial GLM: $\ln\left(\mu \right)=\beta_{0}+\beta_{1}\times G+E$. Where G is equal to 1 if the studied gene belongs to the Gene Set of interest and 0 otherwise. The term E corrects for the intersection bias of the background regions, with E being the expected number of hits for a particular gene: E = $n\;*\;\frac{K}{N}$, with K being the number of query regions, and N the number of background regions. We tested whether $\beta_{1}$ is greater than zero using a Wald Test. The model is fitted using the python statsmodels^53^ library. We considered GO terms with more than 3 genes and less than 1,000, and applied the Benjamini-Hochberg FDR correction. The approach is similar to Chip-Enrich and Poly-Enrich, which has shown that gene-wise modeling is required to reduce false discoveries, but these two methods do not offer a model for our case, where the query regions are a subset of a set of background regions. To improve the readability of the GO enrichments, we identified clusters of GO terms given to similar genes using a graph clustering approach to reduce term redundancy. Starting from a binary matrix with genes as columns and significant (5% FDR) GO terms as rows, we built a nearest neighbor graph of GO terms using the Yule metric. We performed graph clustering on this NN graph and chose the GO term with the smallest p-Value as the cluster representative for each cluster.

#### TF motifs identification

We employed HOMER^28^ 4.11 to identify TF motifs within the HOMER database designed for the hg38 genome. For each of the 51 RNAPII clusters, we conducted a systematic search for known motifs (default parameters), leveraging the extensive collection of motifs available within the HOMER database. The HOMER database encompasses a diverse range of experimentally validated motifs, which have been curated and annotated to provide reliable and accurate motif predictions.

#### GWAS traits and summary statistics

We used Stratified LD-Score Regression^66^ (S-LDSR) to compute enrichments of heritability phenotypes for subgroups of consensus peaks. We downloaded all available GWAS summary statistics files from UK Biobank^67^^,^^68^ (http://www.nealelab.is/uk-biobank), and only kept traits with strong heritability (noted “z7”, as recommended by the documentation for this kind of analysis). RNAPII consensus coordinates were lifted to hg19 (UCSC liftover) which caused 873 RNAPII consensus to be removed. An SNP was assigned to a RNAPII consensus if it overlaps any part of the RNAPII consensus. The LDSC pipeline was run at default settings and Bonferroni correction was applied on the obtained p values. Heritability enrichments are defined as: $\frac{Proportion\;of\;h²}{Proportion\;of\;intersected\;SNPs}$, where h^2^ is the SNP-based heritability (see LD-Score paper^66^).

#### RNA-seq expression quantification of RNAPII-bound regions

We quantified RNA-seq expression on RNAPII-bound regions using featureCounts^69^ similar to RNA-seq gene quantification. Instead of genes as sampling points, RNAPII-bound regions were used and standardised to 1kb long, centered on the consensus centroids (±500bp). Multi-mapping reads were excluded. Given the similarities of our data with scRNA-seq datasets, we employed several methods commonly used in the scRNA-seq field. Our data has a large number of samples (equivalent to cells) with much lower read counts compared to traditional gene-centric RNA-seq experiments. Before conducting each analysis, we preprocessed the data by filtering out RNAP2-bound regions that did not have at least one read count in three samples. This filtering step helped to remove noise and increase the quality of the data. The “gene-centric” GTEx count table was retrieved from GTEx.^15^

#### Count normalisation and transformation

An overview of our count processing is available in Figure S14. Counts were normalised using the scran pooling and deconvolution^70^ approaches, as RNAPII-bound region counts have a large fraction of zeroes causing issues on approaches such as DESeq2’s median of ratios. A small modification of the method was used to compute the size factors only using the top 5% “most detectable” bound regions. We defined detectability as the number of samples that have at least one read at an RNAPII-bound region. To break ties, we computed detectability at 2 reads, 3 reads … up to 5 reads, which is sufficient to break most ties. This helped to reduce the number of non-expressed RNAPII-bound regions or RNAPII-bound regions that are expressed in a single condition only, which should not be considered for an optimal normalisation. This can be seen as something analogous to the use of the geometric mean in DESeq2’s median of ratios, which considers only genes with at least 1 read in each sample. RNAPII counts were transformed using the Pearson residuals of a regularised Negative Binomial (NB) model, similarly to what is used in SCTransform.^71^ This kind of transform has been shown to better discriminate between biological conditions in scRNA-seq experiments, as well as reducing batch effects caused by differences in sequencing depths at small counts. The model for a gene/RNAPII-bound region expression is: ln(μ)=βX+ln(s). Where μ are the predicted means for a bound region/gene for each sample, β are the fitted model coefficients, X is the design matrix, and s are the count normalisation factors for each sample. In this work we use a simple intercept but the model allows more complex experimental designs. We use the following NB(μ, α) variance formulation: $V\left(\mu \right)=\mu +\alpha \;\mu^{2}$. We fitted a trendline of the overdispersion parameter α as a function of the mean, so we obtain a regularised estimate of the variance that only depends on the mean. To do so, we binned genes/RNAPII-bound regions into 20 groups according to their quantile of mean expression, evaluated each gene/bound region overdispersion parameter, find the modal value of overdispersion within a group using a kernel density estimate (with Silverman’s rule to estimate bandwidth), and linearly interpolate results between each group/quantile of mean expression. We only used up to 5000 RNAPII-bound regions/genes to fit the mean/overdispersion relationship to speed up computations. The python statsmodels library was used to fit the NB models with the more robust Nelder-Mead solver instead of BFGS. The pearson residuals are then computed as following: $=\frac{x-\mu }{\sqrt{V\left(\mu \right)}}$ , where x is the count value. A custom python implementation was employed as the SCTransform package failed to run on the RNAPII count matrices, possibly due to much larger counts than UMI scRNA-seq experiments, causing numerical instability when fitting the models. The original implementation clips the pearson residuals at $\pm \sqrt{n/4}$ by default, where n is the number of cells/samples, in order to reduce the influence of outliers. We found these bounds to be quite small when dealing with smaller sample sizes, which can remove biological signals. Instead, we clipped values at $\pm \sqrt{9+n/4}$ , creating larger bounds for small sample sizes without changing the large sample size behavior.

#### Reads distribution on standardised RNAPII consensus

To visualise the read profiles, we employed the following methodology. Initially, we sampled the RNA-seq signal within 10-base pair (bp) windows, which were positioned within the standardised 1-kilobase (kb) RNAPII consensus regions. To integrate the data from multiple samples, we generated a pooled "meta-sample" by summing the number of reads of each sample. Furthermore, we normalised the read counts for each sample by dividing them by the total number of reads, ensuring accurate comparisons across samples. To standardise the windowed signal of each RNAPII consensus region, we normalised it by dividing by the maximum pooled signal across all 10bp windows within that specific RNAPII consensus region. This normalisation step allowed us to eliminate any potential bias and enabled fair comparisons between different regions and datasets. Next, we applied ward hierarchical clustering to arrange the rows of the heatmap for each dataset. This clustering approach facilitated the identification of similar transcriptional patterns. Finally, to generate an overall profile for each dataset, we calculated the mean value of the pooled, maximum normalised transcriptional signal across all RNAPII consensus regions.

#### Unsupervised feature selection, dimensionality reduction and predictive models

Feature selection in scRNA-seq is a common step that allows to remove a large fraction of potentially uninformative bound regions/genes (i.e., those with very low expression or those with ubiquitous expression, which are not informative of the sample/cell biology). Typically, around 2000 to 3000 genes are kept in scRNA-seq experiments, but this number is generally tuned for each experiment. To automatically select “highly variable” features for each dataset, we computed the sum of the squared pearson residuals, which are asymptotically following a $\chi^{2}$ distribution with n - p degrees of freedom, n being the number of samples, and p the number of parameters of the model (1 in our case). We performed an upper tail test for each gene/bound region and kept bound regions at an FDR of 5%. This selects only sufficiently expressed genes above the mean-variance trendline, and due to the clipping of the pearson residuals also removes outliers with an extreme variance (Figure S22A). We performed PCA on the Pearson Residuals of these highly variable features. To automatically identify the optimal number of Principal Components, we used Horn’s Permutation Parallel Analysis, which has been found to be one of the most effective approaches to identify the number of components in factor analysis^72^ (cit). This approach generates row–wise permutations for each feature, computes PCA on these permuted datasets, then the selected number of components is the threshold at which the eigenvalues from the randomised dataset are larger than the real dataset. We performed 3 permutations due to the computational cost of this approach, which is acceptable as the randomised eigenvalues are very stable on large matrices (Figure S22B). We used the fast “randomised” solver from the python sklearn^59^ library to compute PCAs.

For UMAP visualisation, we used 30 neighbors, a min_dist parameter of 0.5, Pearson correlation as the metric and use data in PCA space as input. For heatmaps, we used a similar approach as the RNAPII heatmap, except that the data was used in PCA space as input to the UMAP pass for the samples, and used Pearson correlation as the metric for both samples and RNAPII-bound regions. The predictive model uses a Catboost gradient boosted decision tree model that takes as input the data in PCA space. Default settings were used with the exception of balanced class weights (where each sample is reweighted by class proportion). We used balanced accuracy (where each sample is reweighted by class proportion) as the main metric to evaluate the model over a stratified 10--Fold Cross Validation.

#### Identification of per tissue markers and “meta-clustering”

For each dataset (TCGA, ENCODE, GTEx, RNAPII), we identified markers for each annotation (i.e., Pol2+Liver, GTEx+Blood). To identify markers in the three RNA-seq datasets, we performed a group-versus-rest, one sided t-test on the Pearson Residuals. We kept over-expressed bound regions with log2 Fold Change above 0.25, and detectable in at least 10% or 2+ samples (whichever is the largest). For the RNAPII dataset, we performed an hypergeometric test for each RNAPII consensus, where: N, the population size, is the number of peaks across all experiments; K, the number of successes in the population, is the number of peaks across all experiments with the annotation of interest; n, the number of draws, is the number of experiments that has a peak at the studied consensus peak; k, the number of observed successes, is the number of experiments with the annotation of interest that has a peak at the studied consensus peak. We used a BH FDR cutoff of 5% in both cases. This yields a binary vector which indicates whether a RNAPII-bound region is a marker or not for each dataset+annotation. We removed RNAPII-bound regions which are markers in more than 10% of the dataset+annotation combinations or in less than two dataset+annotation. An Average Linkage clustering using the Yule binary metric was performed, which we found to be less sensitive to the number of identified markers.

#### Tissue specific eQTL enrichments

We downloaded per tissue eQTL data from GTEx “GTEx_Analysis_v8_eQTL.tar” and used the list of significant eQTL-gene pairs for each tissue. For each SNP listed as an eQTL we stored whether it is listed as an eQTL or not in each tissue. SNPs listed as eQTLs in more than 10% of the tissues (6 or more) were removed to keep variants that are likely located in tissue specific regulatory regions. Using the list of per dataset, per tissue marker RNAPII-bound regions, we kept RNAPII-bound regions that are markers in less than 10% of the tissues in a dataset (6 or less) and removed non-marker RNAPII-bound regions. We removed tissues having less than 50 markers left after this step. We computed pairwise intersection enrichment p values between the tissue-specific eQTLs and the marker RNAPII-bound regions (SNP intersection against whole bound region). We computed an hypergeometric enrichment p value for each of these intersections as following: N, the population size, is the number of RNAPII-bound regions (after filtering); K, the number of successes in the population, is the number of RNAPII-bound regions intersecting an eQTL from the eQTL-wise tissue of interest; n, the number of draws, is the number of marker RNAPII-bound regions of the second tissue of interest; k, the number of observed successes, is the number of marker RNAPII-bound regions of the second tissue of interest intersecting an eQTL from the eQTL-wise tissue of interest.

#### Differential expression

To identify differentially expressed (DE) RNAPII-bound regions between tumor and normal tissues, we performed a t-test on the Pearson Residuals with an FDR cutoff of 5%. We constrained DE RNAPII-bound regions to have an absolute log2 Fold Change above 0.25, and to be detectable in at least 10% or 2+ samples (whichever is the largest) of either class (normal/tumor). For the detection of Tumor Subtype specific markers, we compared the expression of samples of a subtype to reference normal samples. We used the same significance cutoffs. We considered a marker to be subtype-specific only if it appeared for this subtype. Linear modeling methods such as DESeq2 ran out of memory on large datasets and required an excessive computation time. A t-test was used to accommodate large datasets (100+ samples x 180 000 bound regions in most datasets) and to keep an uniform processing for each dataset for our cross-dataset analyses. With sufficiently large sample sizes, the t-test yields robust markers, although with less statistical power (Figures S22C and S22D). To evaluate our approach on a much smaller dataset with less statistical power, we selected samples from two similar types of heart tissues from GTEx and downsampled to obtain an n = 3 comparison. Here, we used DESeq2^73^ to maximise statistical power. We performed 100 random sampling iterations to obtain 3 samples for each tissue, evaluated DE in each iteration, then kept bound regions supported as DE in at least half of the downsampling iterations. To evaluate the relationship between sample size and statistical power, we performed 10 downsampling iterations for each sample size. GREAT^65^ v4.0.4 analyses were performed on BRCA TNBC specific RNAPII-bound regions, identifying enrichment of the “MAP kinase tyrosine/serine/threonine phosphatase activity” GO:0017017 term (Binom FDR Q-Val 1.34e-14), with TNBC specific RNAPII-bound regions associated with 10 DUSPs genes.

#### Using GTEx normal tissues instead of TCGA normal tissue

We evaluated the variation of cancer-specific intergenic RNAPII markers when using GTEx normal tissues instead of TCGA normal tissues. We selected tumor samples from TCGA and utilised GTEx normal samples as the reference group (Figure S23). Differential expression analysis was performed between these two groups, employing the same methodology as in the primary TCGA cancer vs. TCGA normal analysis. The RNAPII consensus regions were then ranked based on their p values, and an equal number of markers were retained as in the main analysis. To evaluate the overlap between the TCGA normal vs. GTEx normal analyses, we computed two statistics: A) the recall, which represents the fraction of shared markers, and B) the fold change of the observed number of shared markers compared to the expected value if they were chosen randomly. It is important to note that the original TCGA normal vs. TCGA tumor samples were not paired in the analysis (there are, in fact, more cancer samples than normal ones). Additionally, GTEx and TCGA employ different protocols for tissue sampling, conservation, and sequencing, which introduces a noticeable batch effect. Our findings indicate a substantial and statistically significant overlap between the two analyses, with the exception of ESCA (Esophageal carcinoma) cancer, which also exhibited lower classification accuracy in machine learning.

#### Identification of pan-cancer markers

To identify RNAPII-bound regions whose expression is associated with survival or DE (separately) in tumor tissues in multiple cancers (“pan-cancer markers”), we randomly selected for each cancer the same number of marker bound regions as observed in this cancer. This process was repeated 100 times to obtain the expected distribution of the number of cancers in which a bound region is a marker. We identified the “pan-cancer threshold” as the threshold where less than 5% of the observed markers are expected to belong to the null distribution (equivalent to 5% FDR, see Figure S24). This approach allowed us to set a statistically meaningful threshold to identify bound regions that are markers in more cancers than expected at random, instead of an arbitrary threshold.

#### Survival analysis

For survival analysis, we fit a linear Cox Proportional Hazards regression model on the Pearson residuals using the Python Lifelines library, and use a 5% FDR threshold. Kaplan-meier survival curves were created using the kaplanmeier python library. The maxstat R library was used to obtain the optimal expression cutpoint as well as the associated maximally selected log -rank statistic p value (using the most accurate “condMC”′ method with 100 000 samples to compute the p value).

#### Enrichment of cancer hallmark-related genes

To investigate the enrichment of cancer hallmark-related genes, we utilised a gene set enrichment analysis (GSEA) approach. The list of candidate genes associated with specific cancer hallmarks was obtained from the CHG database (http://www.bio-bigdata.com/CHG/) which provides a comprehensive collection of genes with verified and putative links in various cancer-associated biological processes. We conducted GSEA analysis on RNAPII markers genomic regions. However, instead of utilising Gene Ontology (GO) terms, we employed the cancer hallmarks as defined in the CHG database. Cancer hallmarks encompass key biological processes and pathways that contribute to tumorigenesis and cancer progression. We assessed the enrichment of cancer hallmark-related genes nearby the pan-cancer RNAPII markers. This analysis allowed us to identify potential associations between the RNAPII marker and specific cancer hallmarks, providing insights into the functional relevance of these regions in cancer biology.

## Data Availability

RNA polymerase II ChIP-seq data are publicly available in NCBI-GEO, and data accessions for ChIP-seq are listed in Table S1. The GTEx^15^ eQTL data were obtained from GTEx v8. Human regulatory TF catalog was obtained from ReMap 2022 release.^19^ ENCODE RNA-seq raw sequencing data (Accession IDs in Table S4) are available at https://www.encodeproject.org/. TCGA and GTEx RNA-seq raw sequencing data are available under controlled access to ensure appropriate data usage. Access to these protected data must be requested through the dbGaP portal. The Cancer Genome Atlas^16^ (TCGA) RNA-seq BAM files are accessible through dbGaP under accession no. phs000178.v11.p8.c1 (TCGA) and at NCI’s Genomic Data Commons (http://gdc.cancer.gov) under project TCGA. Genotype-Tissue Expression (GTEx) RNA-seq BAM files are accessible through dbGaP under accession no. phs000424.v8.p2.c1 (GTEx) and at the GTEx portal (https://gtexportal.org/home/). Identified and annotated RNAPII consensus are available in Data S1. We deposited the codes and bioinformatics environments in GitHub at https://github.com/benoitballester/Pol2Atlas. The processed data matrices and files can be accessed on Zenodo.^18^^,^^52^ Both data and codes are publicly available for the replication of the whole study.

## Supplemental information

Document S1. Figures S1–S24

Table S1. RNAPII ChIP-seq dataset annotation, related to Figures 1 and 2

Table S2. ENCODE accession IDs for ATAC and H3K27ac, related to STAR Methods

Table S3. Tissue names for simplified annotation, related to Figures 2

Table S4. RNA-seq ENCODE dataset annotation, related to Figures 4 and 5

Data S1. RNAPII consensus in BED format, related to STAR Methods This is a BED 9 format, a commonly used tab-delimited file format in genomics and bioinformatics. It is primarily used to represent genomic interval data, such as gene annotations, DNA binding sites, and other genomic features. The format comprises nine mandatory columns, each providing specific information about the genomic region being described. Col 1 Chromosome: The name of the chromosome where the RNAPII consensus is located. Col 2 Start: The starting position of the RNAPII consensus, using a 0-based coordinate system (i.e., the first position is labeled as 0). Col 3 End: The ending position of the RNAPII consensus. Col 4 Name: The nomenclature corresponds to the regulatory genome annotation linked to the respective RNAPII consensus (e.g., Enhancer-like), followed by the corresponding biotype (e.g., Breast). Several genomic annotations may be linked to a single RNAPII consensus (combined using "/"). A biotype can be succeeded by "?" or "??", signifying situations where the biotype association from the Figure 3B clusters and GO enrichment remains uncertain (due to manual curation). Col 5 Score: A numerical value representing the number of aggregated RNAPII peaks. Col 6 Strand: Not stranded ".". Col 7 Thick Start: The starting position of the feature’s "thick" region, used to display the highest point that aggregate the RNAPII consensus. Col 8 Thick End: The ending position of the feature’s "thick" region. Col 9 Item RGB: A comma-separated triplet of red, green, and blue color values (0–255) following Figure 1E colors convention.

Document S2. Article plus supplemental information
