# Supplementary material for: Characterizing intergenic transcription at RNA polymerase II binding sites in normal and cancer tissues
Source: Cell Genom. 2023 Sep 29;3(10):100411. doi: 10.1016/j.xgen.2023.100411 (PMC10589727; doi:10.1016/j.xgen.2023.100411)
Supplement: Document S1. Figures S1–S24 [file mmc1.pdf]

Cell Genomics, Volume *3*

Supplemental information

Characterizing intergenic transcription

at RNA polymerase II binding sites

in normal and cancer tissues

Pierre de Langen, Fayrouz Hammal, Elise Guéret, Jean-Christophe Mouren, Lionel Spinelli, and Benoit Ballester

SUPPLEMENTAL INFORMATION

Characterising intergenic transcription at RNA polymerase II binding sites in normal and cancer tissues

Figure S1 : Characteristics of the RNAPII atlas, related to STAR Methods, related to STAR Methods 2
Figure S2 : Examples of intergenic RNAPII occupancy in MCF-7 and HEK293 cells, related to Figure 1 3
Figure S3 : Large scale integration methodology of RNAPII ChIP-seq, related to STAR Methods 4
Figure S4 : Intergenic RNAPII bound regions are co-localizing with regulatory elements, related to Figure 1 5
Figure S5 : Intergenic RNAPII bound regions are enriched in end of gene regulatory elements, related to STAR Methods 6
Figure S6 : Comparison of intergenic RNAPII consensus with genomic resources, related to Figure 1 7
Figure S7 : Genome-wide large scale integration of 890 Human H3K27Ac Histone ChIP-seq experiments, related to STAR Methods 8
Figure S8 : RNAPII occupancy clustering and cluster annotation statistics, related to STAR Methods 9
Figure S9 : Tissue-specific biological characteristics of RNAPII consensus, related to Figure 3 10
Figure S10 : Tissue-specific epigenetic states of RNAPII consensus, related to Figure 3 11
Figure S11 : RNAPII-bound regions captures a majority of intergenic transcriptional signal, related to Figure 4 12
Figure S12 : Transcriptional profiles of intergenic RNAPII-bound regions, related to STAR Methods 13
Figure S13 : Flowchart of the RNA-seq pipeline, related to STAR Methods 14
Figure S14 : Intergenic transcription by itself is sufficient to characterise biological conditions, related to STAR Methods 15
Figure S15 : Tissue-specific regulatory variants are enriched within tissue-specific Intergenic transcripts, related to STAR Methods 16
Figure S16 : The intergenic transcriptional signal is not driven by end-of-gene transcription, related to STAR Methods 17
Figure S17 : Differentially expressed RNAPII-bound regions can be detected at smaller sample sizes, related to STAR Methods 18
Figure S19 : Non coding transcription captured at RNAPII-bound regions discriminates normal and tumour tissues, related to Figure 6 20
Figure S20: Non coding transcription captured at RNAPII-bound regions is prognostic of the patient's survival, related to Figure 7 21
Figure S21: Supplementary multi-cancer prognostic marker, related to Figure 7 22
Figure S22 : Mean-variance trendline, Feature selection and PCA Permutation Parallel analysis, related to STAR Methods 23
Figure S23 : Using GTEx normal tissues instead of TCGA normal tissue, related to STAR Methods 24
Figure S24 : Distribution of differentially expressed RNAPII-bound regions in cancers, related to STAR Methods 25

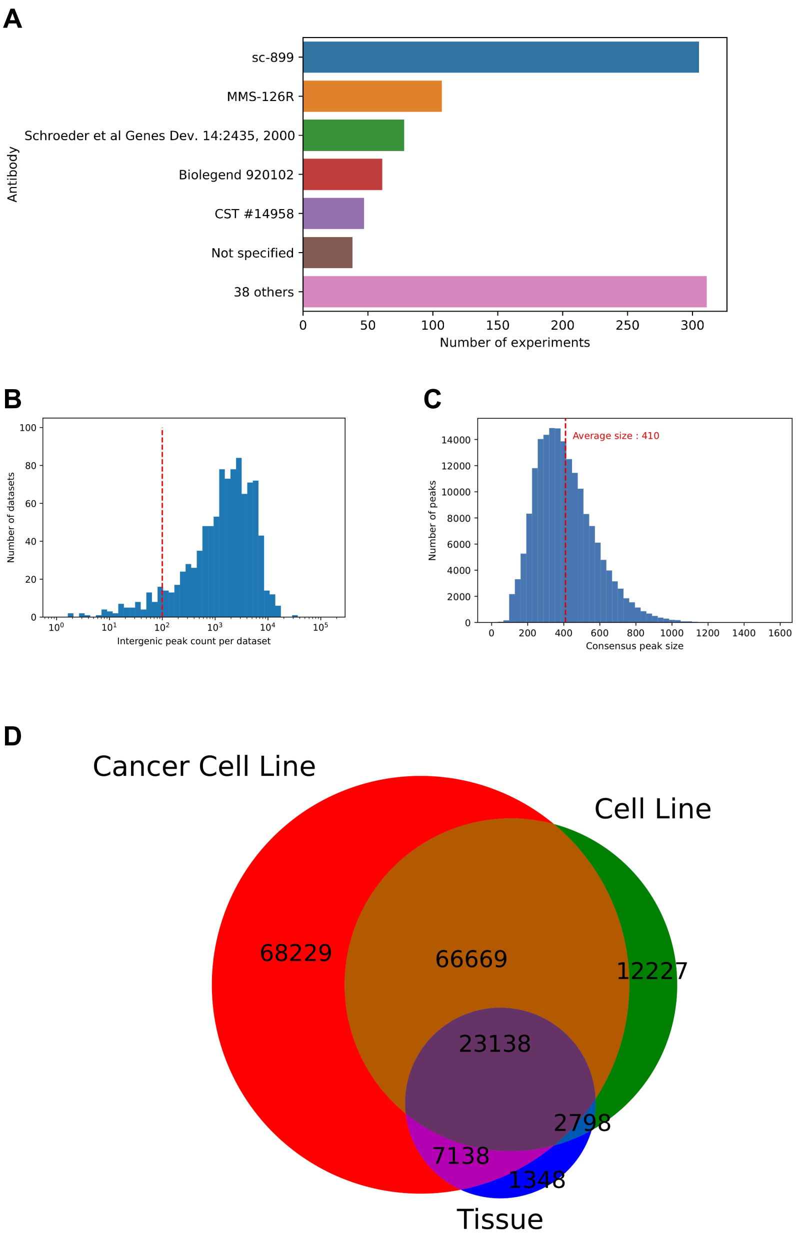

Figure S1 : Characteristics of the RNAPII atlas, related to STAR Methods, related to STAR Methods

A. Number of RNAPII ChIP-seq experiments in which each antibody is used. **B.** Histogram of the distribution of the number of Intergenic RNAPII peaks per dataset, average 2,787 intergenic binding events per dataset. Red line indicates minimal cutoff for a dataset to be retained. **C.** Histogram of the distribution of the intergenic consensus peaks sizes, with an average consensus width of 410bp (red line). **D.** Detectability of RNAPII consensus peaks per sample category ; Cancer cell lines, Cell lines and Tissues.

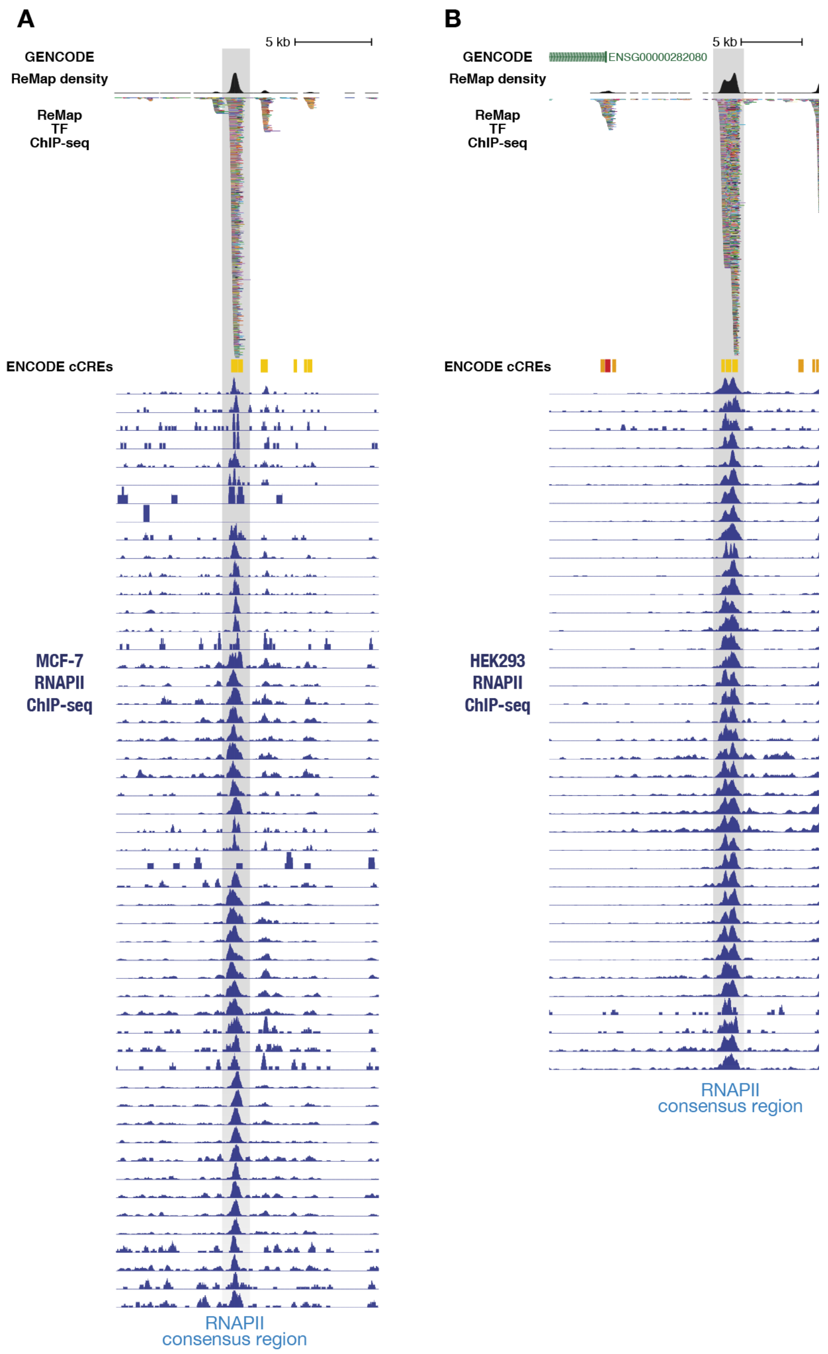

Figure S2 : Examples of intergenic RNAPII occupancy in MCF-7 and HEK293 cells, related to Figure 1

A. Genomic example on chr1:31170514-31171424, displaying RNAPII raw ChIP-seq signals across MCF-7 cell lines (Blue) at the location of a RNAPII consensus region (grey bar), with GENCODE. ReMap TF ChIP-seq and ENCODE cCREs tracks. **B.** Similarly, a second example of RNAPII ChIP-seq signals at chr12:8023634-8024204 with HEK293 cell lines.

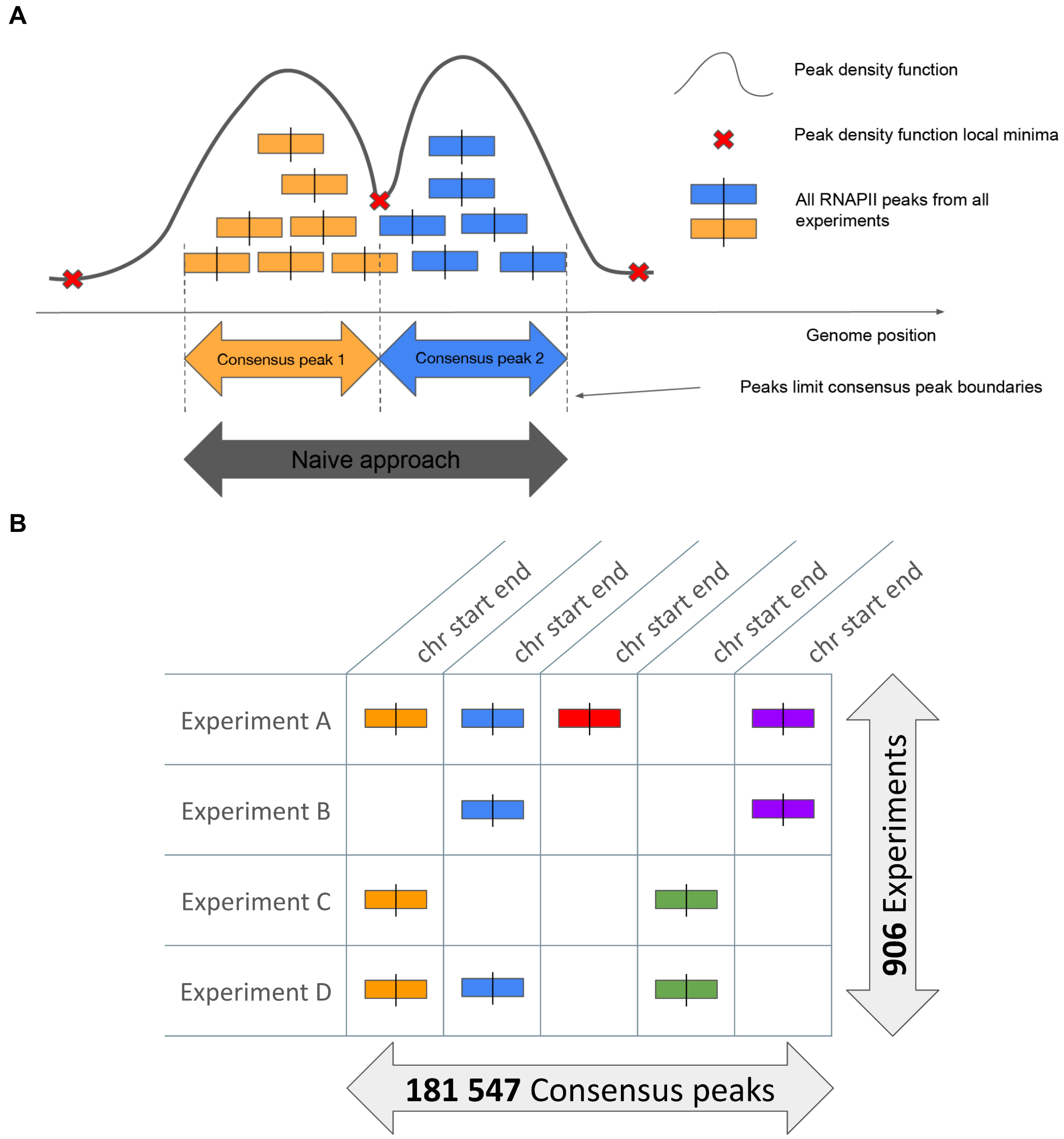

Figure S3 : Large scale integration methodology of RNAPII ChIP-seq, related to STAR Methods

Schematic of our post peak-calling integration methodology : **A.** Identification of consensus peaks via a peak density-based approach. Naive approach refers to a simple merge on overlap. **B.** Summarization of all datasets / consensus peaks in a binary matrix storing the presence or absence of RNAPII at each consensus peak in each experiment.

A

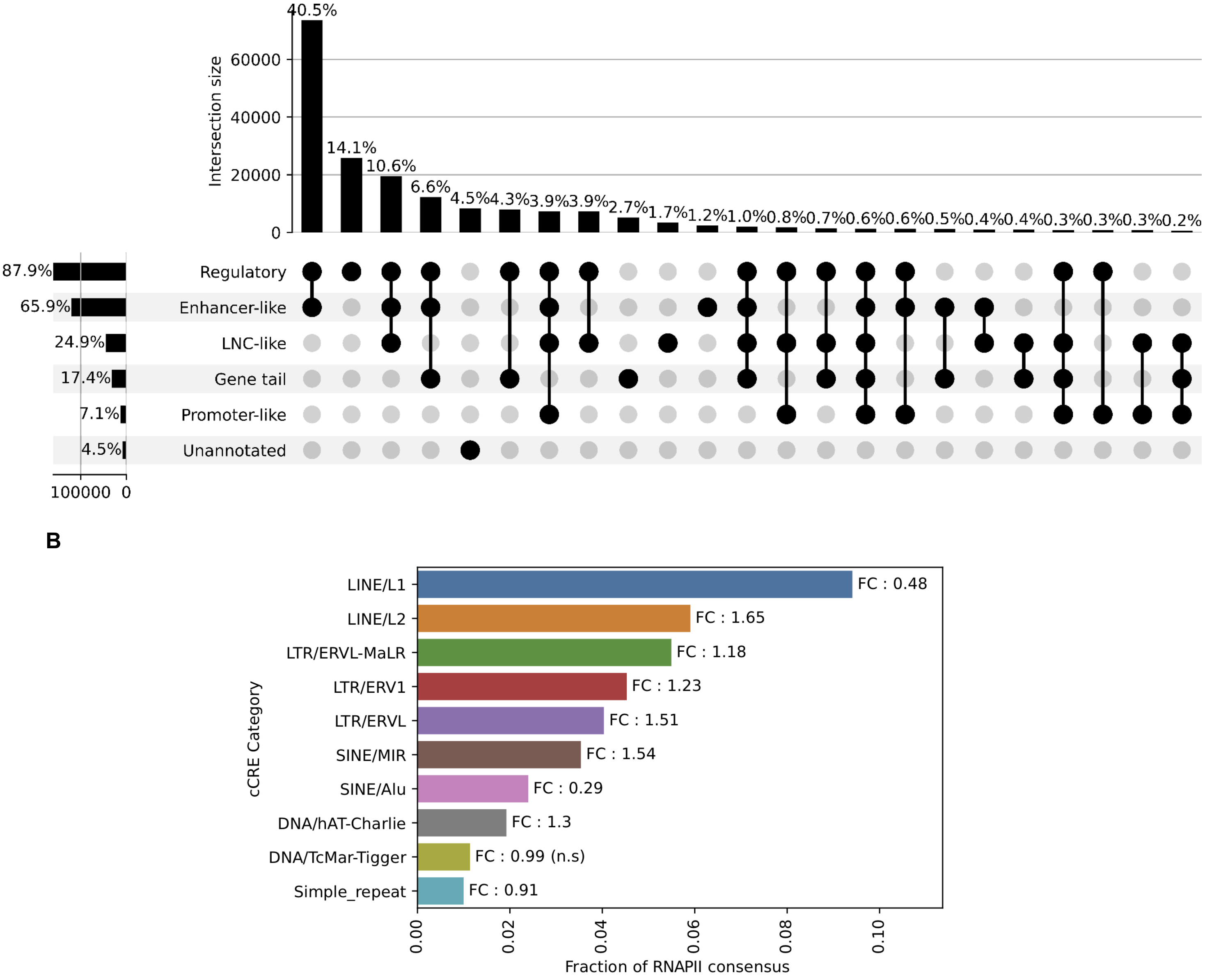

Figure S4 : Intergenic RNAPII bound regions are co-localizing with regulatory elements, related to Figure 1

A. UpSet plot of putative functional annotations of intergenic RNAPII consensus, derived from reference databases (see methods for details). Rare combinations with intersection sizes smaller than 0.1% of all intergenic RNAPII consensus have been removed for clarity. **B.** Fraction of consensus peaks intersected for the top ten most intersected repeat families. FC corresponds to fold change enrichment versus random regions. All Fold Changes are statistically significant (FDR < 0.05) unless otherwise mentioned (hypergeometric test, see methods, $p < 1e-100$).

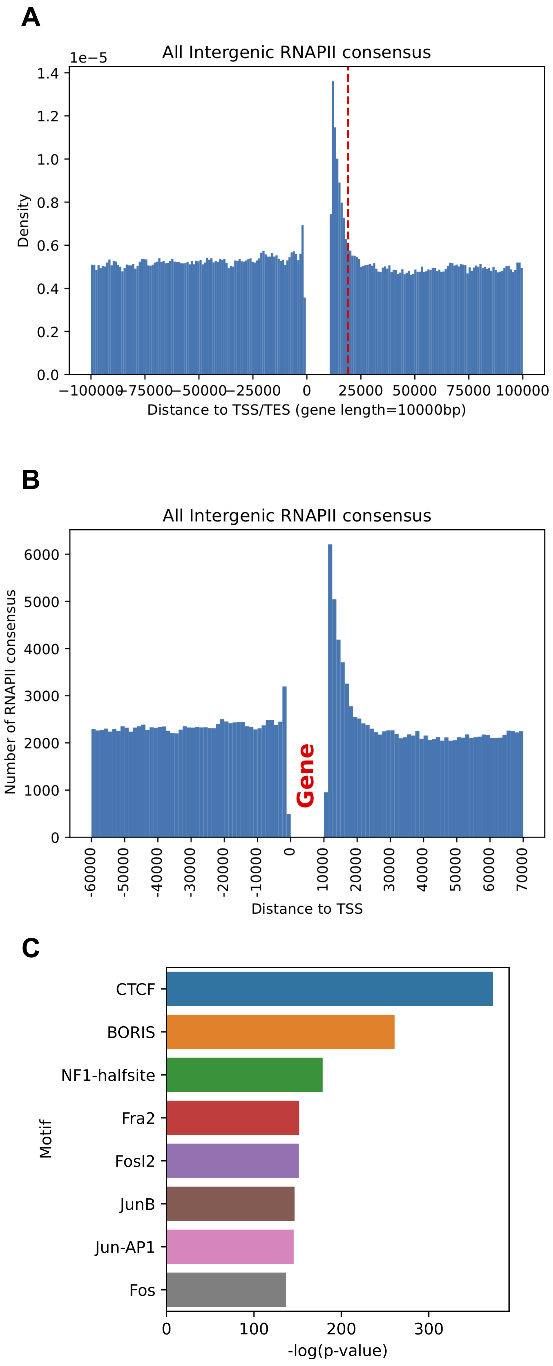

Figure S5 : Intergenic RNAPII bound regions are enriched in end of gene regulatory elements, related to STAR Methods

A. Distributions of RNAPII consensus centroids relative to protein coding genes (gene length standardised to 10kb), red line indicates 9kb after Transcription End Site (TES). **B**. Distribution of RNAPII consensus counts by 5kb windows relative to TSS of and TES of protein coding genes (red), in narrower genomic coordinates. **C.** HOMER top 10 known motif enrichment -log10(p-values) for RNAPII consensus within 9kb of a TES. The top 2 TFs are CTCF and CTCFL (BORIS).

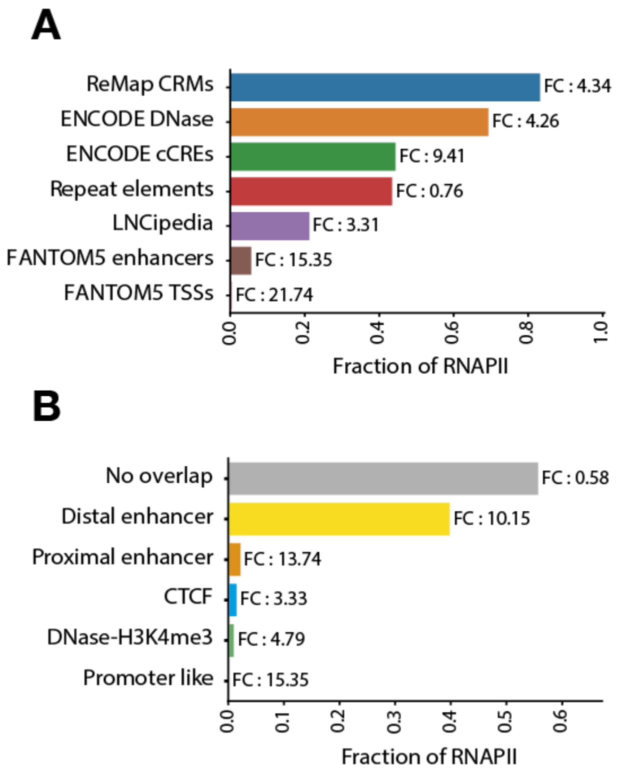

Figure S6 : Comparison of intergenic RNAPII consensus with genomic resources, related to Figure 1

A. Comparison of RNAPII consensus location with major genomic resources of regulatory and non-coding elements. **B.** Distribution of candidate cis-Regulatory Elements (cCREs) derived from ENCODE compared to the RNAPII atlas. FC denotes fold change versus random intergenic regions; all results are strongly statistically significant (hypergeometric test, see methods, $p < 1e-300$).

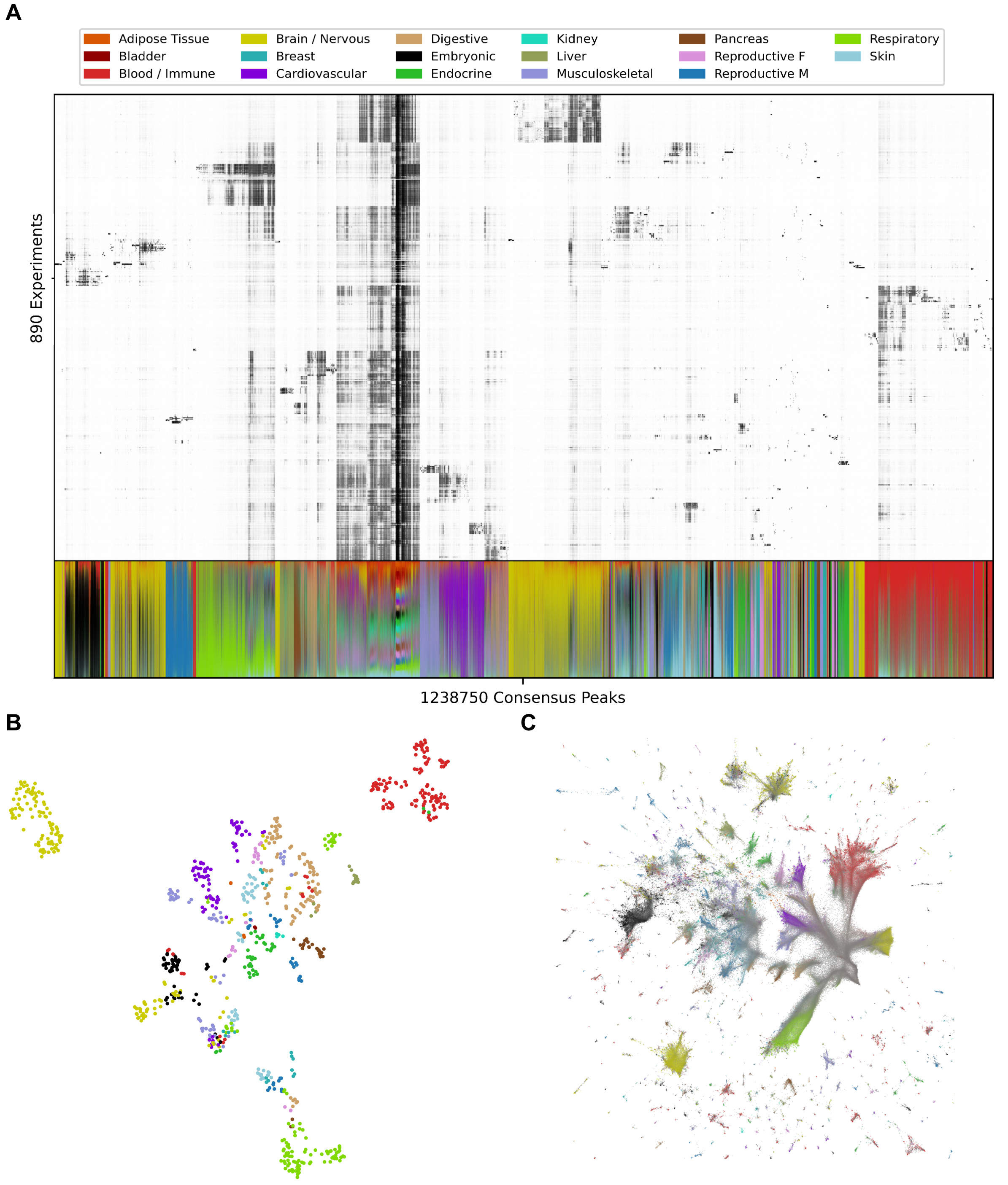

Figure S7 : Genome-wide large scale integration of 890 Human H3K27Ac Histone ChIP-seq experiments, related to STAR Methods

A. H3K27Ac occupancy in 1,238,750 consensus regions across 890 biosamples. Lower panel indicates the normalised contribution of a biotype, in terms of peaks, to each consensus. **B.** Two-dimensional Uniform Manifold Approximation and Projection (UMAP) projection of all 890 H3K27Ac ChIP-seq datasets. **C.** UMAP projection of all H3K27Ac consensus according to their binding patterns, coloured by dominant biotype.

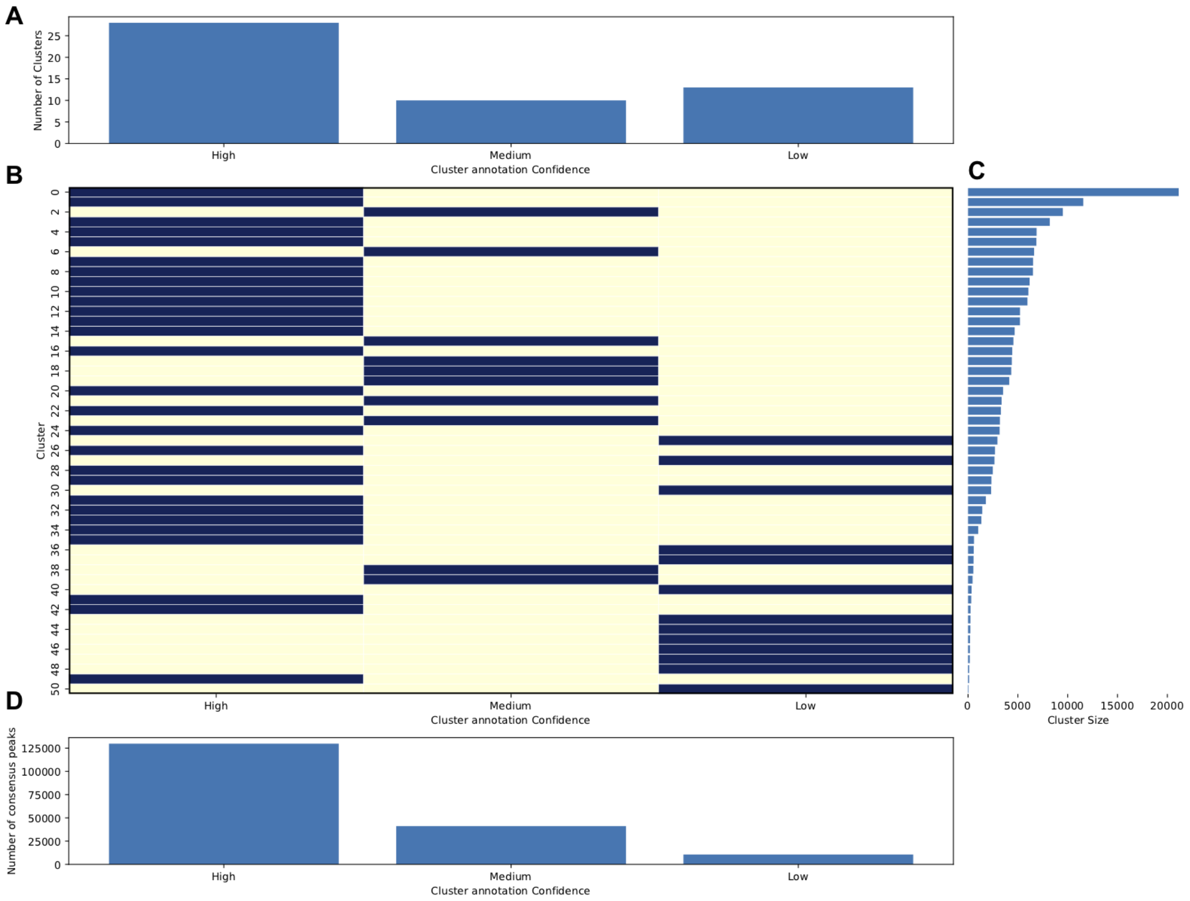

Figure S8 : RNAPII occupancy clustering and cluster annotation statistics, related to STAR Methods

A. Number of clusters by level of annotation confidence (High, Medium, Low), derived with concordance with biological enrichments. **B.** Annotation confidence by cluster (dark = selected annotation). **C.** Number of RNAPII consensus peaks within one of the 51 clusters of annotation **D.** Number of RNAPII consensus peaks across the three levels of annotation confidence.

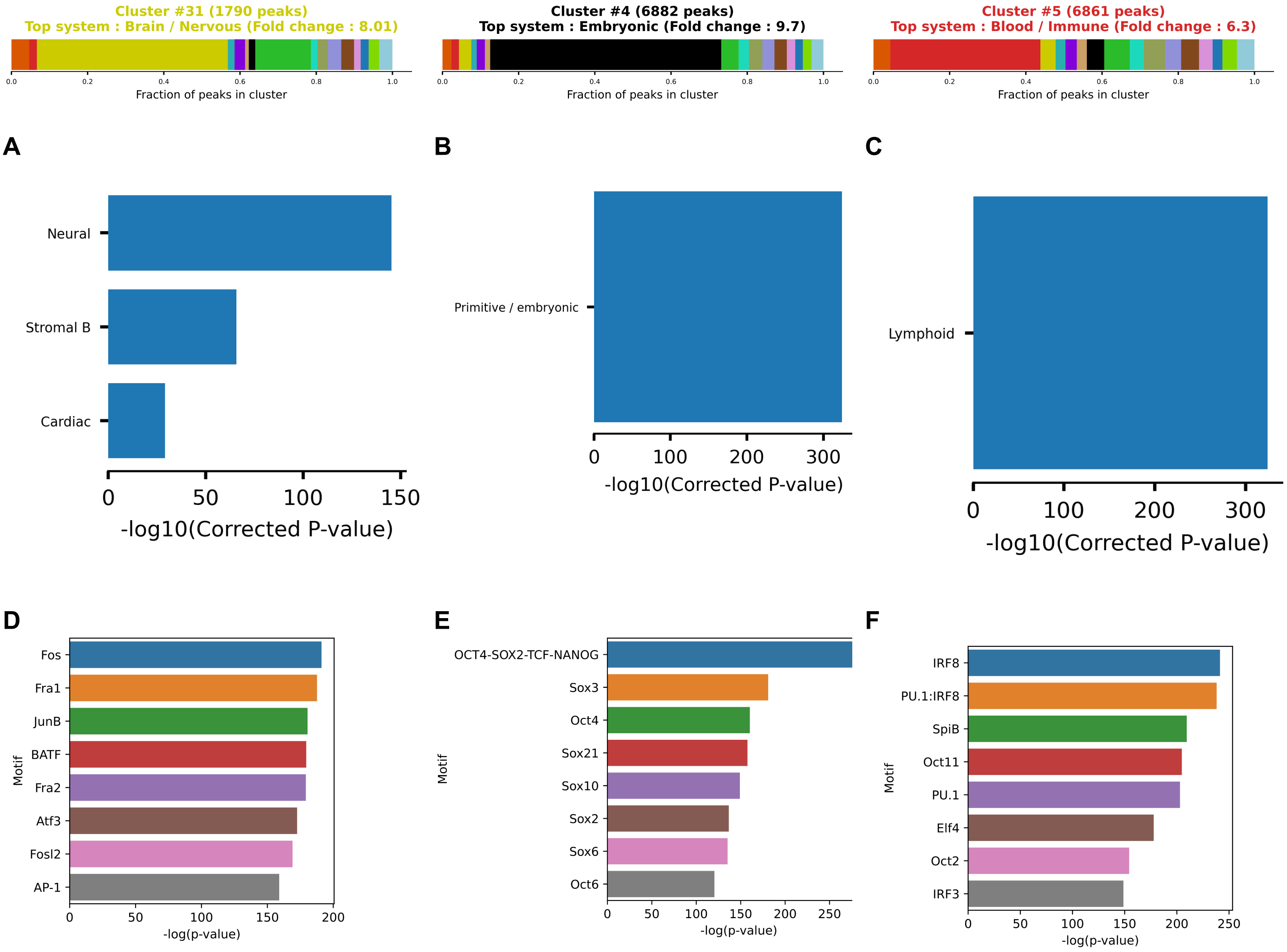

Figure S9 : Tissue-specific biological characteristics of RNAPII consensus, related to Figure 3

Detailed characteristics of three selected RNAPII clusters (#31, #4, #5) compared to the biological classification of the human index of DNase I hypersensitive sites (DHSs) (A,B,C) and HOMER top 8 known DNA motifs enrichments. **A.** RNAPII cluster #31 defined as Brain/Nervous biotype is enriched in Neural, Stromal B and Cardiac DHS. **B.** RNAPII cluster #4 defined as Embryonic biotype is enriched in Primitive/Embryonic DHSs. **C.** RNAPII cluster #5 defined as Blood/Immune biotpype is enriched in Lymphoid DHSs. **D.** The HOMER top 8 DNA motifs for the RNAPII cluster #31 defined as Brain/Nervous biotype contains Fox and Jun. **E.** The RNAPII cluster #4 defined as Embryonic biotype is enriched in Oct4, Sox2 and Nanog DNA motifs. **F.** The RNAPII cluster #5 defined as Blood/Immune biotpype is enriched in IRF8, Pu.1 DNA motifs.

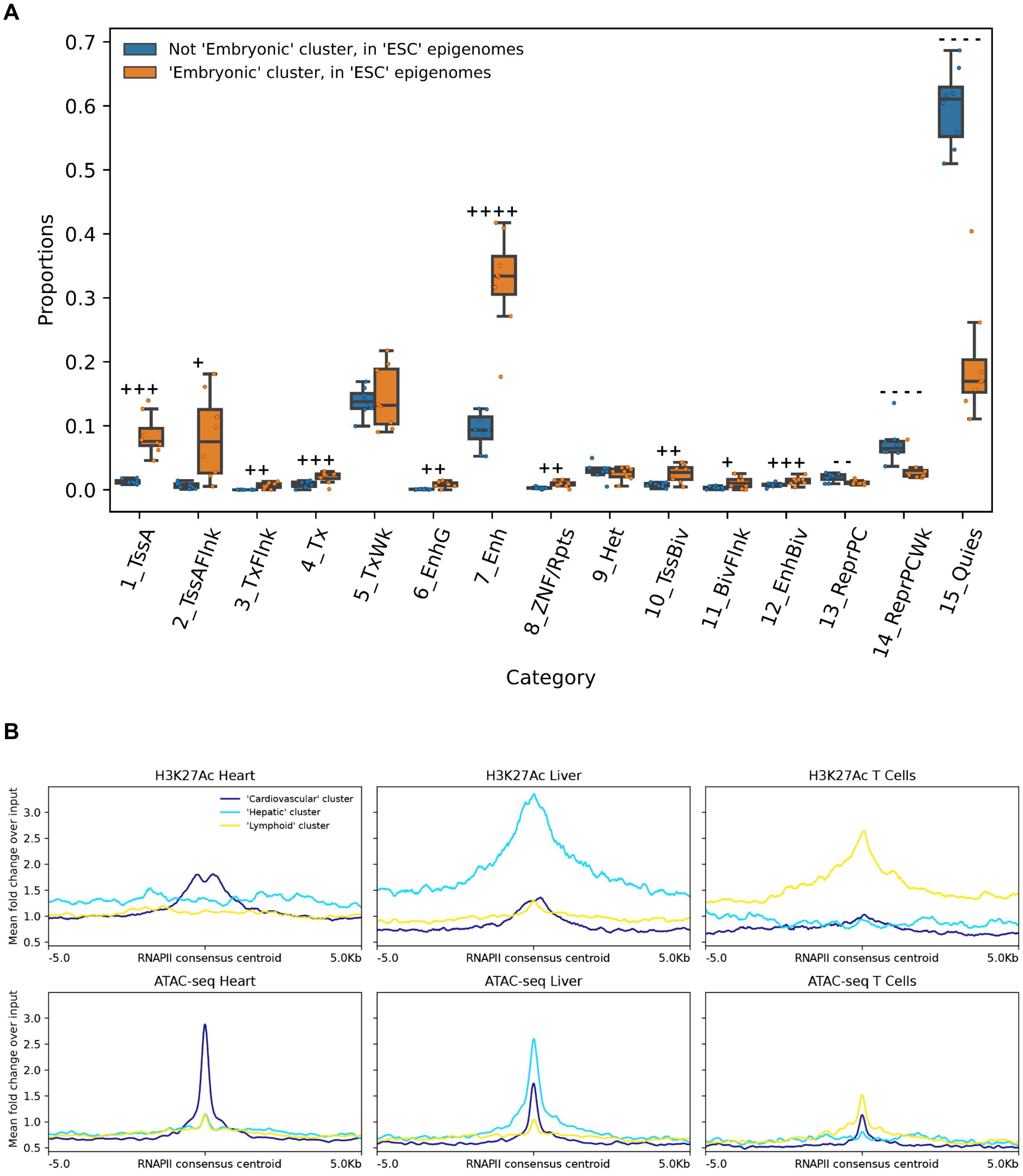

Figure S10 : Tissue-specific epigenetic states of RNAPII consensus, related to Figure 3

A. Proportions of ChromHMM epigenetic states in the "Embryonic" cluster against other Intergenic RNAPII consensus. (* : $p < 0.05$, ** : $p < 0.01$, *** : $p < 0.001$, **** : $p < 0.0001$; + or - indicate sign of mean difference; two-sided paired t-test). Blue boxes correspond to RNAPII peaks not included in the Embryonic cluster #4, but overlapping ESC epigenomes. Orange boxes correspond to RNAPII peaks forming the Embryonic cluster #4, and overlapping ESC epigenomes. **B.** H3K27Ac ChIP-seq and ATAC-seq profiles at clusters of RNAPII consensus. Selected clusters are respectively #5, #10 and #20 for "Cardiovascular", "Hepatic" and "Lymphoid" (largest representative clusters, see supplementary data).

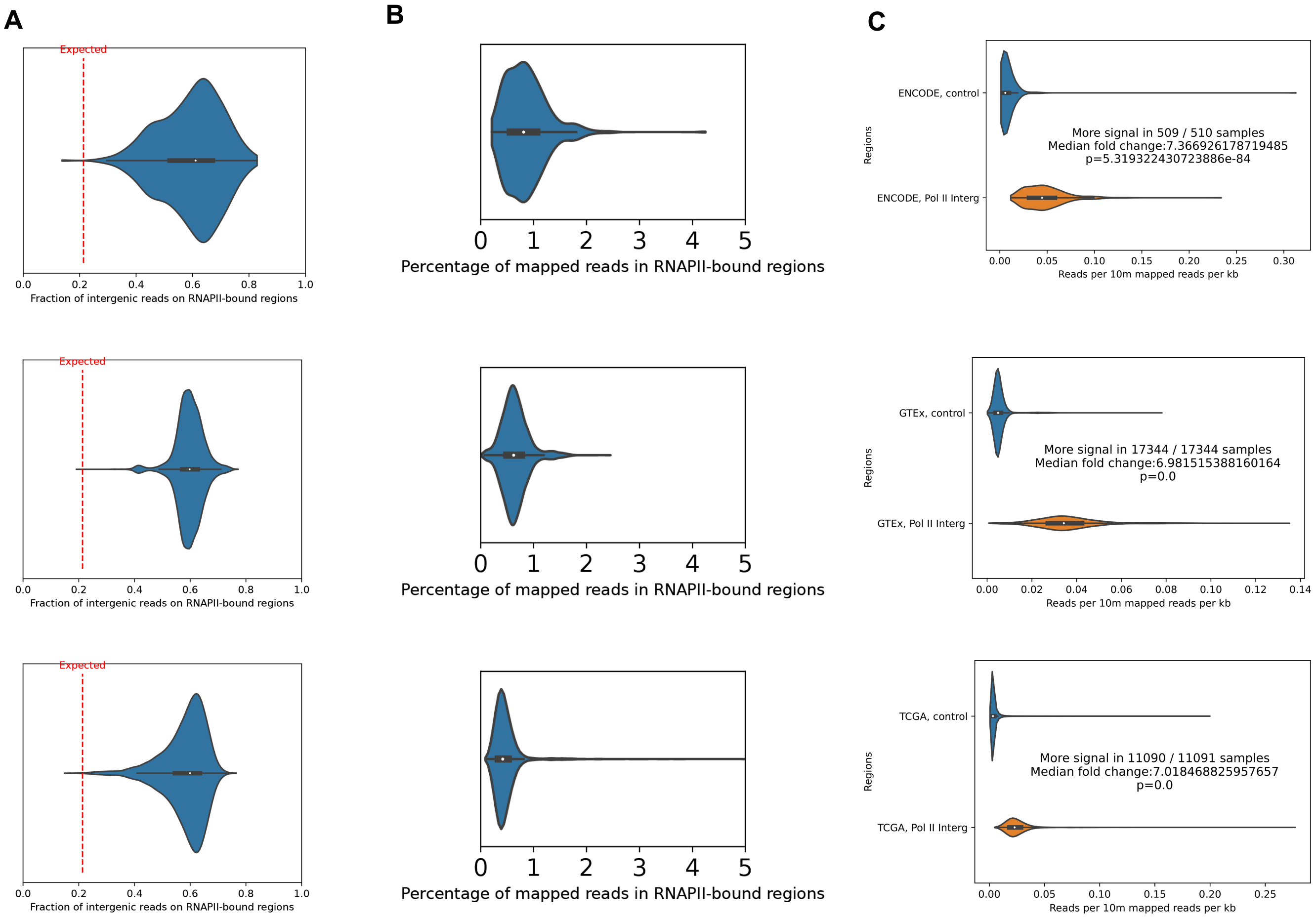

Figure S11 : RNAPII-bound regions captures a majority of intergenic transcriptional signal, related to Figure 4

A. Distribution as violin plots of the fraction of intergenic reads captured by RNAPII-bound regions across samples. Dashed line indicates expected value due to RNAPII-bound regions coverage. From top to bottom : ENCODE, GTEx, TCGA RNA-seq datasets. **B.** Distristribution of the percentage of mapped reads captured by RNAPII-bound regions. **C.** Distribution of the average number of reads per RNAPII-bound region (blue) or control regions (orange) (non RNAPII-bound region, all 1kb binned intergenic regions), across samples (p-values from Mann-Whitney U-test).

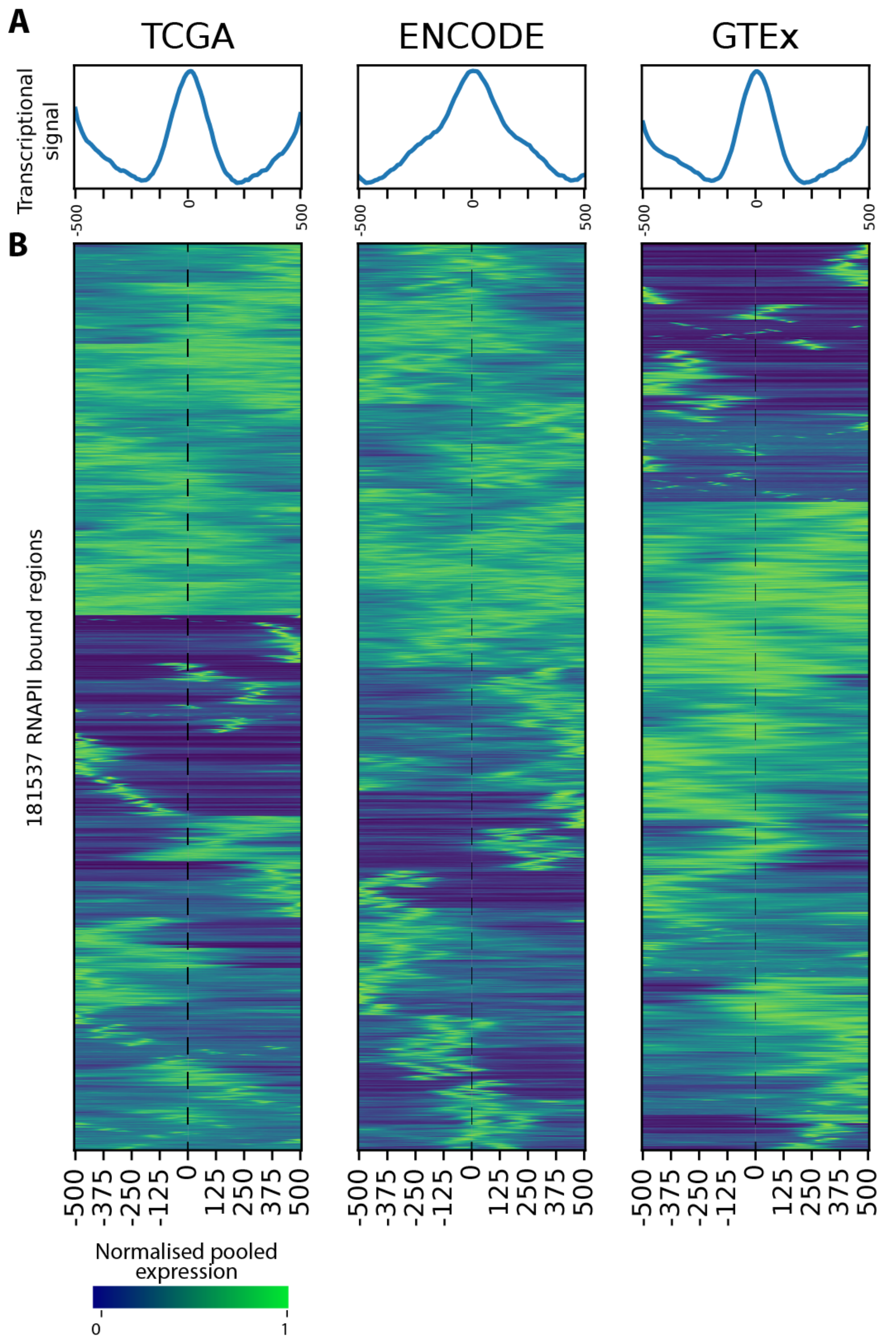

Figure S12 : Transcriptional profiles of intergenic RNAPII-bound regions, related to STAR Methods

A. Pooled, normalised, and averaged transcriptional profiles of intergenic RNAPII-bound regions (methods). **B.** Pooled, normalised, and clustered heatmaps depicting the transcriptional profiles of the 184,547 intergenic RNAPII-bound regions. Each heatmap clustering is performed independently for TCGA, ENCODE, and GTEx datasets.

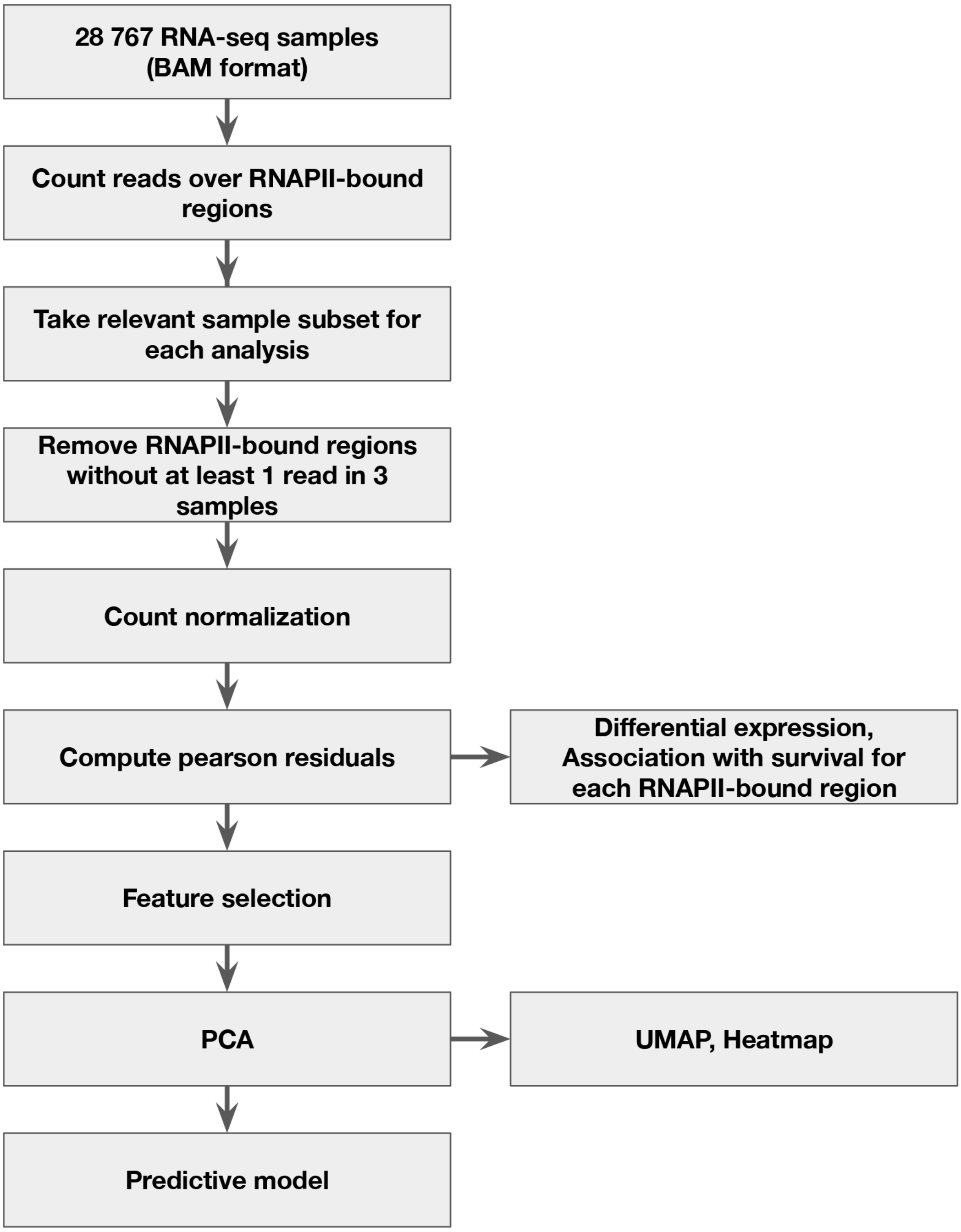

Figure S13 : Flowchart of the RNA-seq pipeline, related to STAR Methods
Simplified schematic of the RNA-seq processing pipeline. See methods for additional details.

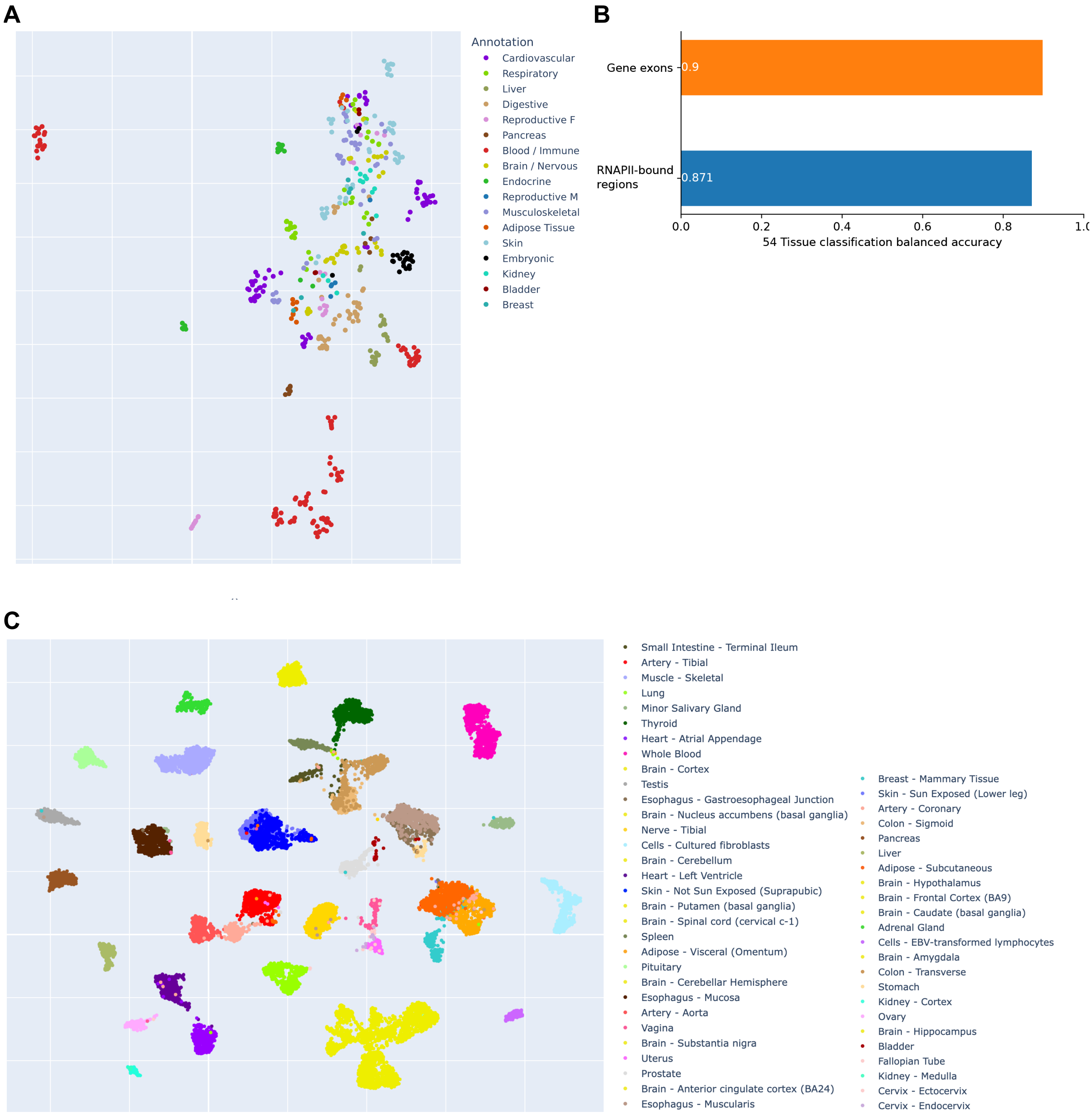

Figure S14 : Intergenic transcription by itself is sufficient to characterise biological conditions, related to STAR Methods

A. UMAP of ENCODE total RNA-seq samples using RNA-seq signal at RNAPII-bound regions. **B.** KNN (5 NN, Pearson correlation as metric) classification balanced accuracy using either Gene expression or RNAPII signal as input. **C.** UMAP of GTEx RNA-seq samples using RNA-seq signal at genes exons. Displayed colours correspond to the official GTEx Tissue colouring conventions.

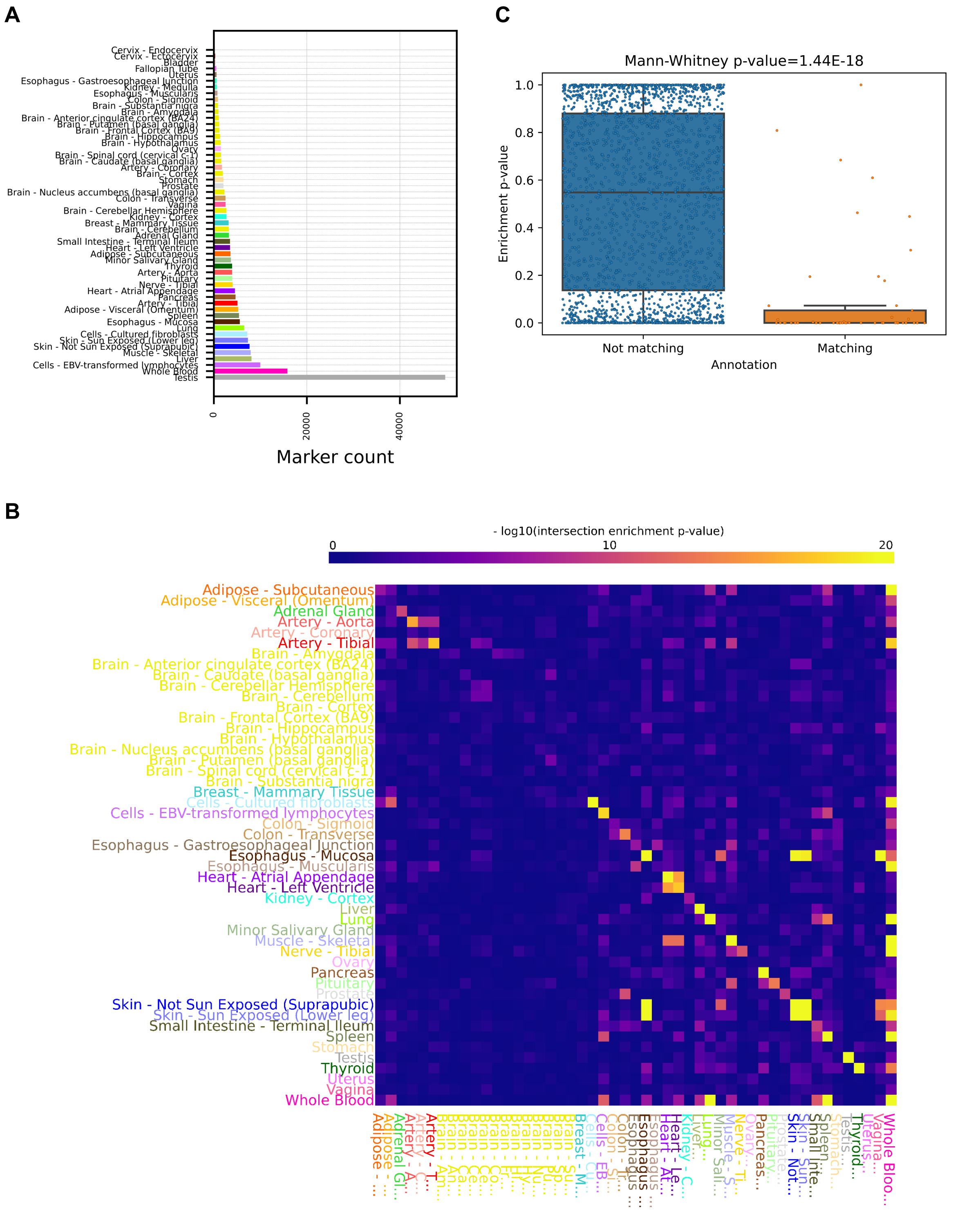

Figure S15 : Tissue-specific regulatory variants are enriched within tissue-specific Intergenic transcripts, related to STAR Methods

A. Number of overexpressed RNAPII-bound regions per GTEX tissue. **B.** Intersection enrichment heatmap between tissue-specific eQTLs (rows) and tissue-specific RNA-seq over-expressed marker RNAPII-bound regions. **C.** Distributions of tissue-matching (i.e. “Artery - Aorta” vs “Artery - Aorta”) and non-matching (i.e. “Artery - Aorta” vs “Artery - Coronary”,“Liver”...) enrichment p-values between tissue-specific eQTLs (rows) and tissue-specific RNA-seq over-expressed markers.

A

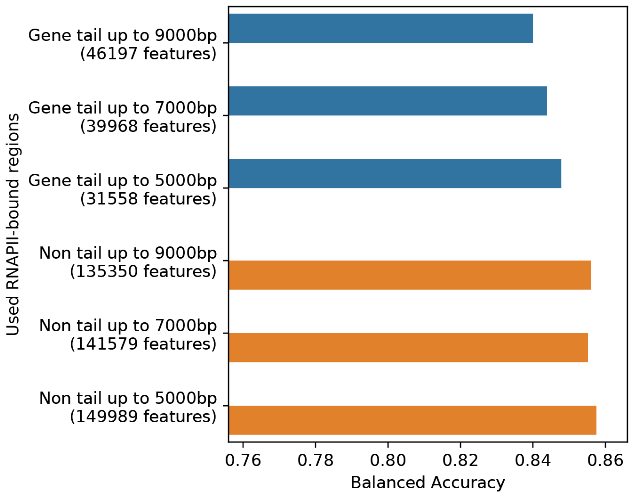

B

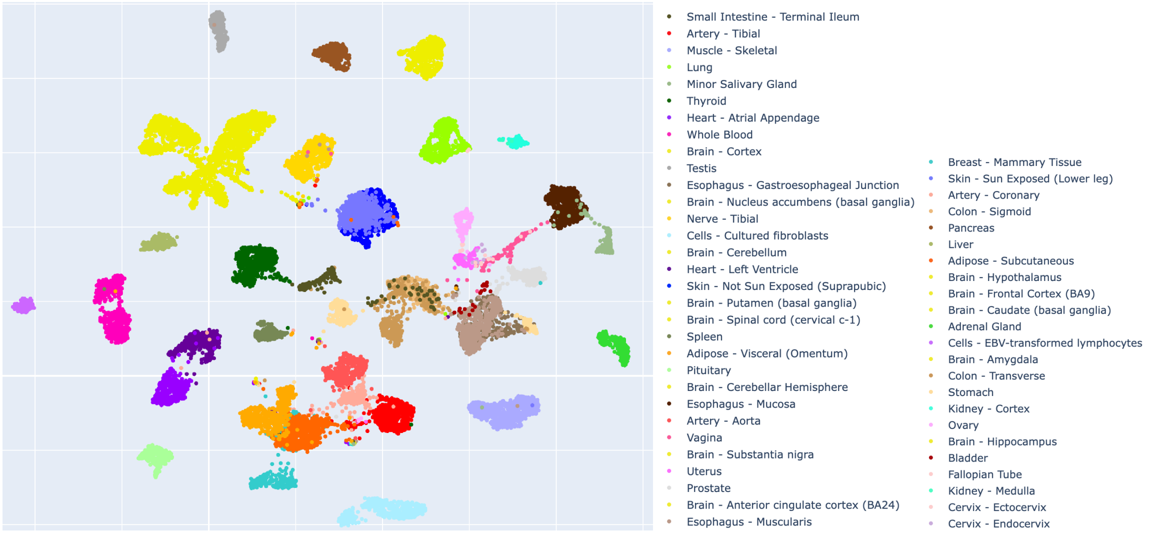

Figure S16 : The intergenic transcriptional signal is not driven by end-of-gene transcription, related to STAR Methods

A. KNN (5 NN, Pearson correlation as metric) classification balanced accuracy using different subsets of intergenic RNAPII-bound regions for classification. **B.** UMAP of GTEx RNA-seq samples using RNA-seq signal at intergenic RNAPII, excluding those located at less than 9,000bp of a Transcription End Site. Displayed colours correspond to the official GTEx Tissue colouring conventions.

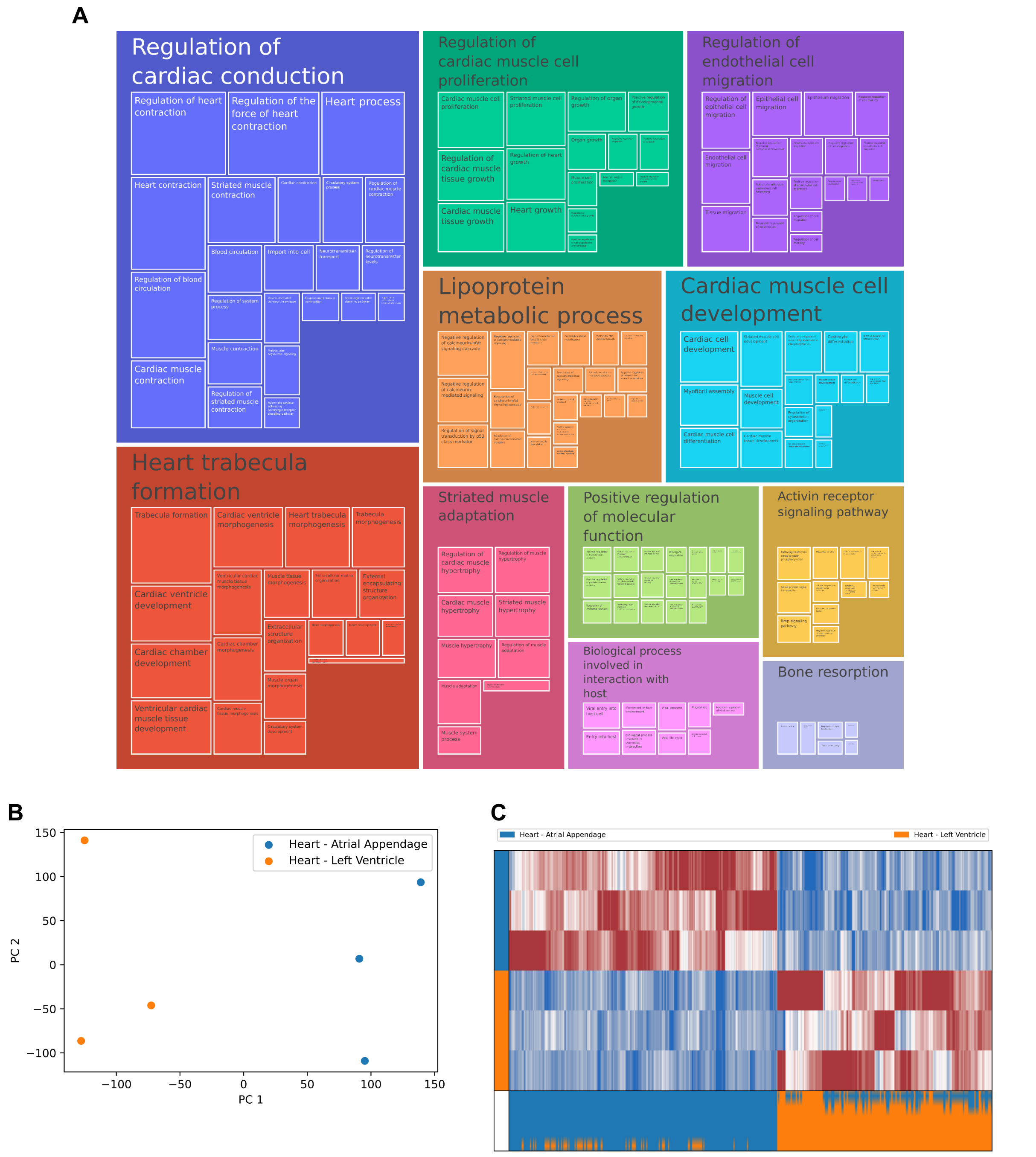

Figure S17 : Differentially expressed RNAPII-bound regions can be detected at smaller sample sizes, related to STAR Methods

A. Clustered GO terms enrichments for genes nearby RNAPII-bound regions differentially expressed between 'Heart - Atrial Appendage' and 'Heart - Left Ventricle' tissues from GTEx in a downsampled n=3 comparison (methods). **B.** First two principal components of RNAPII-bound region expression in a downsampled n=3 comparison. **C.** Heatmap of the Pearson residuals of DE RNAPII-bound regions in a n=3 comparison.

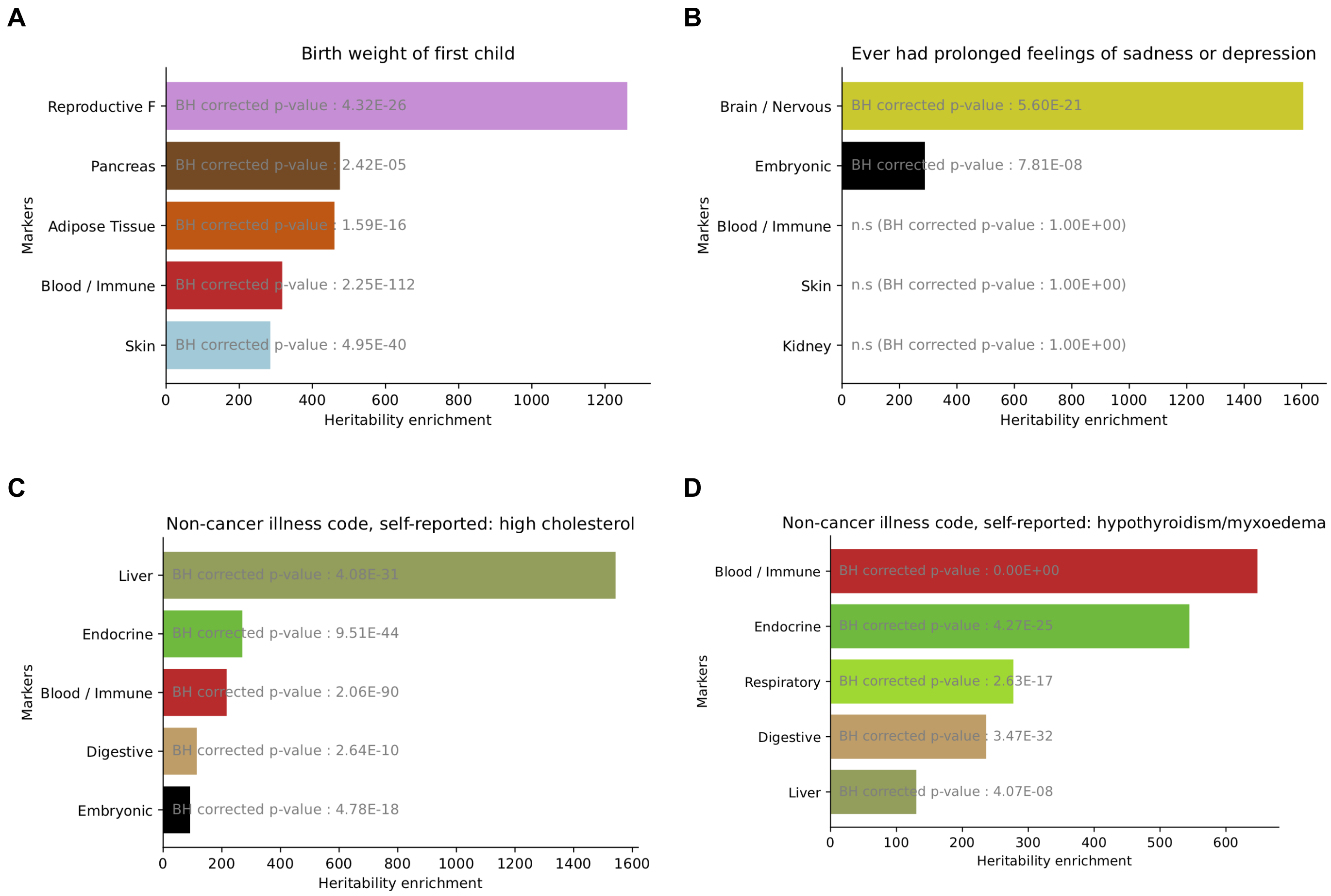

Figure S18 : Per-biotype robustly over-expressed markers display meaningful disease-associated heritability enrichments, related to Figure 5

Top 5 heritability enrichment over robust tissue-specific over-expressed markers (Methods), for 4 disease-associated UK Biobank GWAS traits. Benjamini-Hochberg corrected LD-score regression p-values are indicated and detailed for each trait - marker group pair.

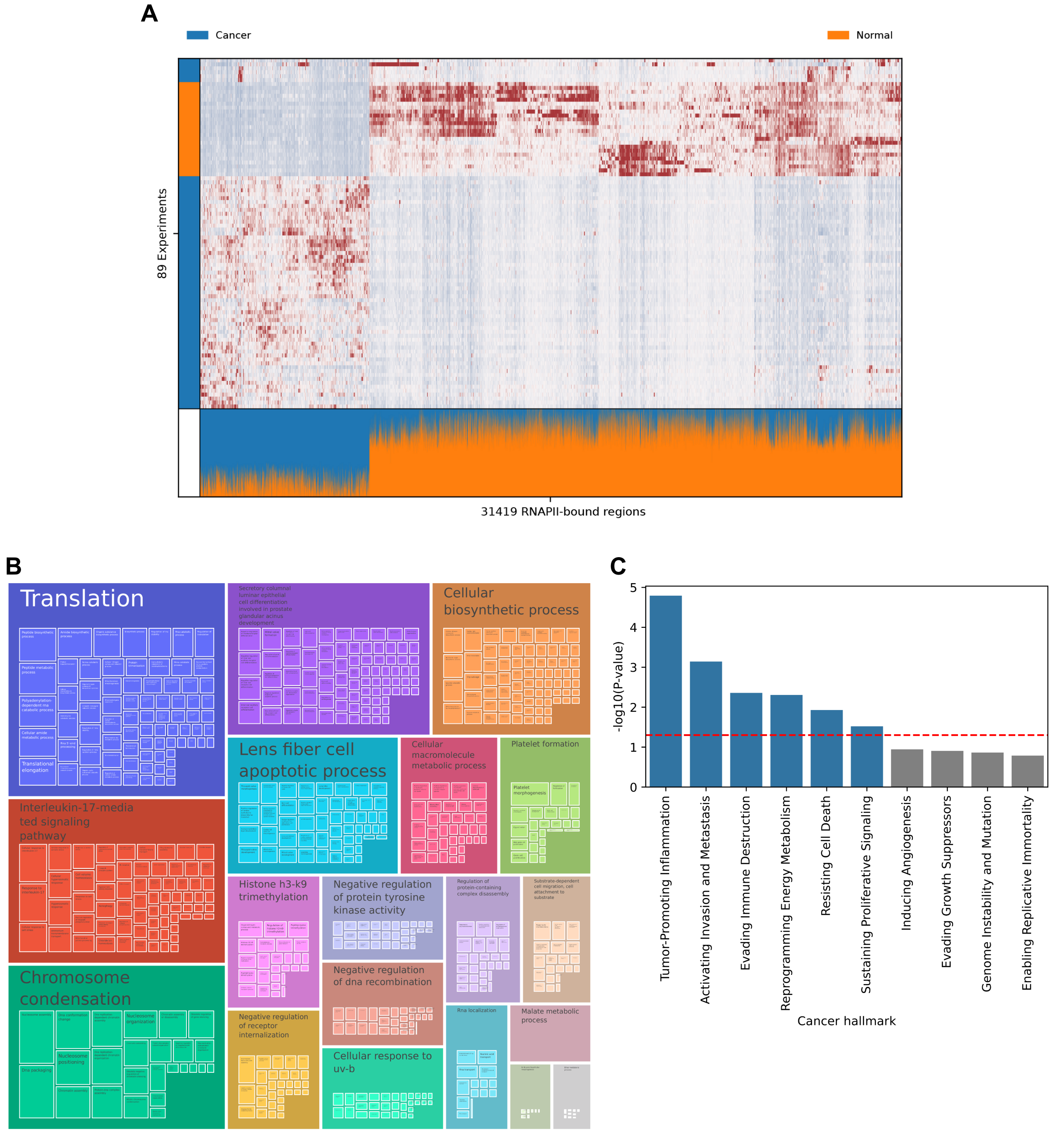

Figure S19 : Non coding transcription captured at RNAPII-bound regions discriminates normal and tumour tissues, related to Figure 6

A. Heatmap of Pearson residuals (clipped at +-3) of DE RNAPII-bound regions in the Kidney Chromophobe Carcinoma dataset (KICH). Red = strongly expressed. Bottom part of the heatmap represents the fraction of normalised reads belonging to either class in each RNAPII-bound region (weighted by class imbalance). **B.** Clustered GO terms enrichments for genes nearby RNAPII-bound regions DE in 7 or more cancers (see methods). All enriched terms are statistically significant (FDR < 0.05, Wald test, see Star Methods). **C.** Gene set enrichment of cancer hallmark genes for genes nearby RNAPII-bound regions DE in 7 or more cancers (see methods). Red dashed line indicates 0.05 p-value threshold.

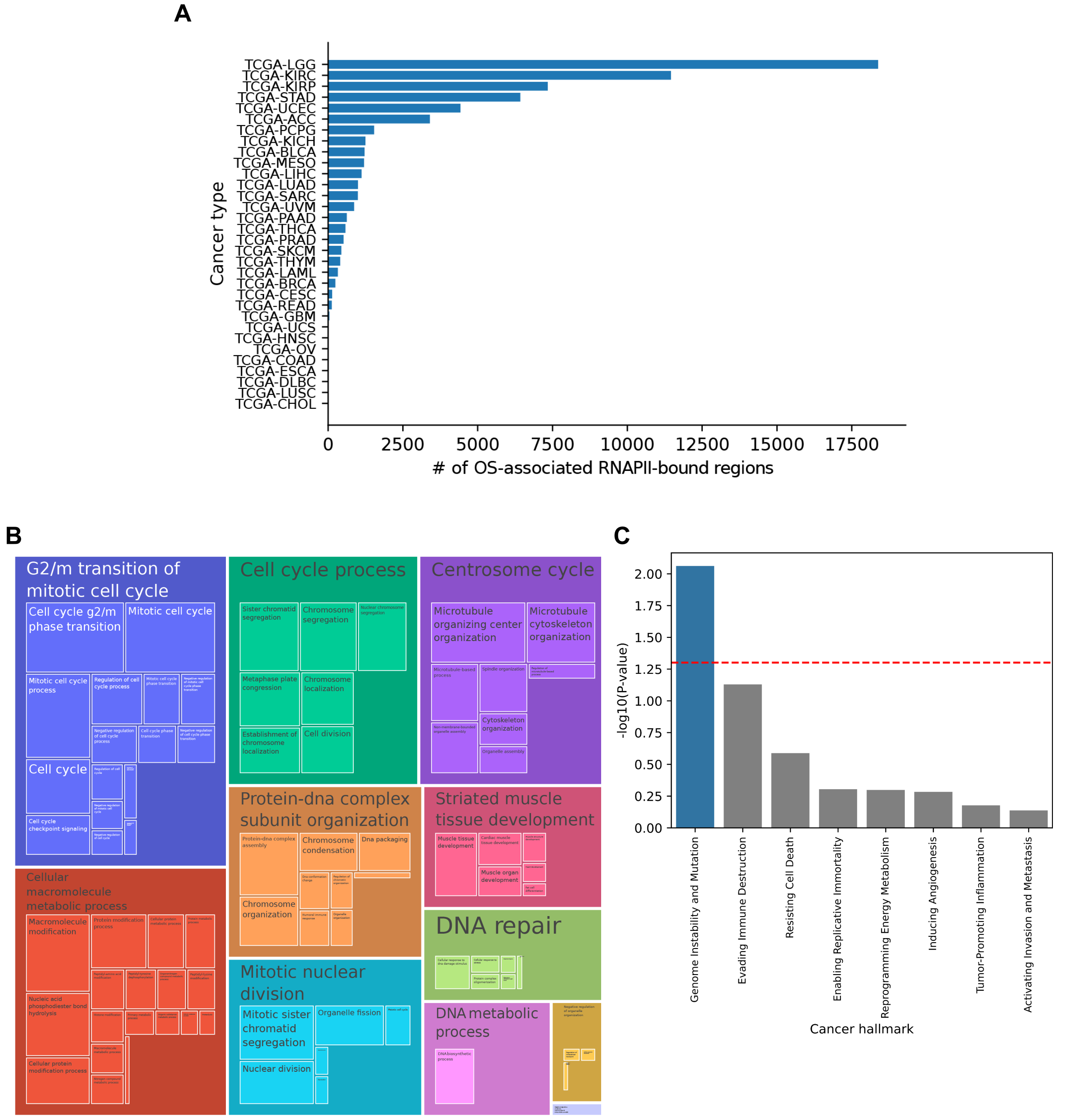

Figure S20: Non coding transcription captured at RNAPII-bound regions is prognostic of the patient's survival, related to Figure 7

A. Number of OS-associated RNAPII-bound regions for each TCGA cancer. **B.** Clustered GO terms enrichments for genes nearby RNAPII-bound regions OS-associated in 5 or more cancers (see methods). **C.** Gene set enrichment of cancer hallmark genes for genes nearby RNAPII-bound regions OS-associated in 5 or more cancers (see Methods). Red line indicates 0.05 p-value threshold.

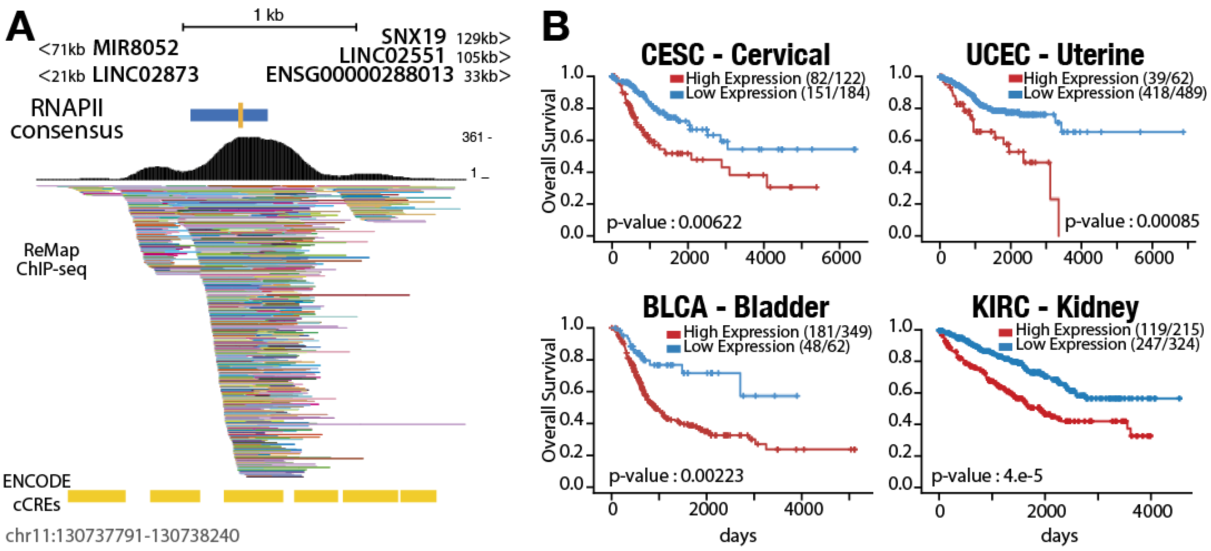

Figure S21: Supplementary multi-cancer prognostic marker, related to Figure 7

A. Genomic landscape of an identified multi-cancer prognostic marker blue bar at chr11:130737791-130738240, located upstream (21kb min) and downstream (33kb min) of non-coding and coding genes. Yellow bars indicate candidate Cis Regulatory Elements (cCREs, Enhancer distal) and ChIP-seq binding from ReMap. This region is located between two non-coding LincRNAs (LINC02873 and ENSG00000288013) at 21kb and 33kb respectively. It is closest to the coding gene SNX19, which encodes a sorting nexin located at 128kb. SNX19 has not been directly linked to cancer survival, other SNXs family members have shown potential prognostic value in various cancers. Decreased expression of SNX1 has been associated with overall survival in colorectal cancer, and down-regulation of SNX2 leads to drug resistance in lung cancer. **B.** Kaplan-Meier survival analysis of Cervical, Uterine, Bladder and Kidney TCGA cancer patients with high (red) and low (blue) expression from the intergenic RNAPII bound region in A. This RNAPII-bound region reveals a previously unknown correlation between its expression and survival in cervical, uterine, and breast cancers (**Figure 7D**).

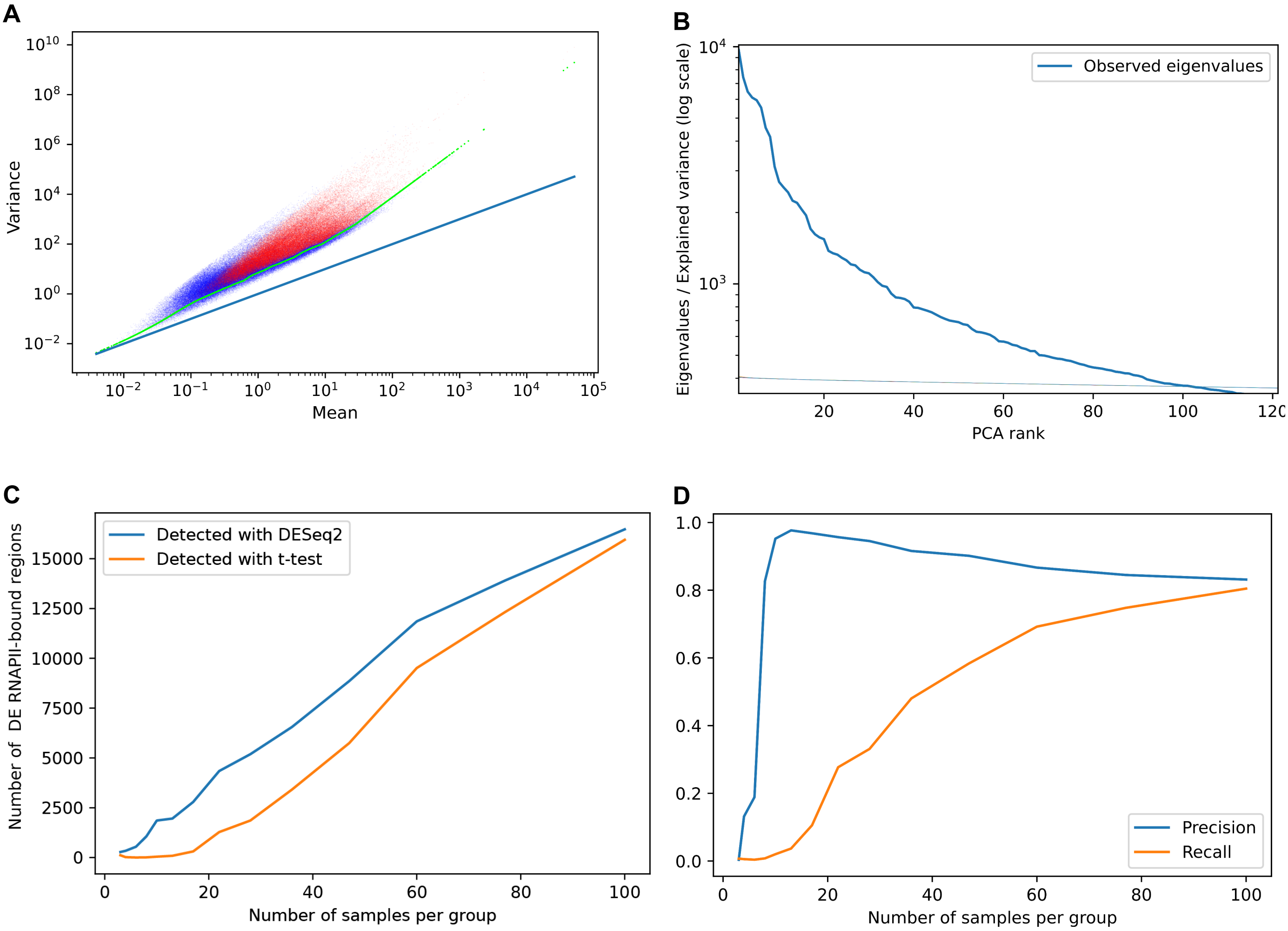

Figure S22 : Mean-variance trendline, Feature selection and PCA Permutation Parallel analysis, related to STAR Methods

A. Scatterplot of the mean and variance of the normalised counts of the 181,547 RNAPII-bound regions in the ENCODE RNA-seq dataset. Red dots are selected “highly variable” RNAPII-bound regions, green dots represent the fitted mean-variance trendline. Blue line is a Poisson mean-variance relationship. **B.** Observed eigenvalues (or explained variance) for each component of the PCA performed on the pearson residuals of selected RNAPII-bound regions in the ENCODE RNA-seq dataset. 100 thinner lines (stacked on the graph) are corresponding to the PCA eigenvalues of each of the 100 permutations of the dataset. Here, only the 102 first components are retained. **C.** Average (over 10 downsampling iterations) number of detected DE RNAPII-bound regions between the two heart tissues in function of the number of samples per group. **D.** Average (over 10 downsampling iterations) precision and recall of the t-test in function of the sample size, using DESeq2 DE-bound regions as a reference.

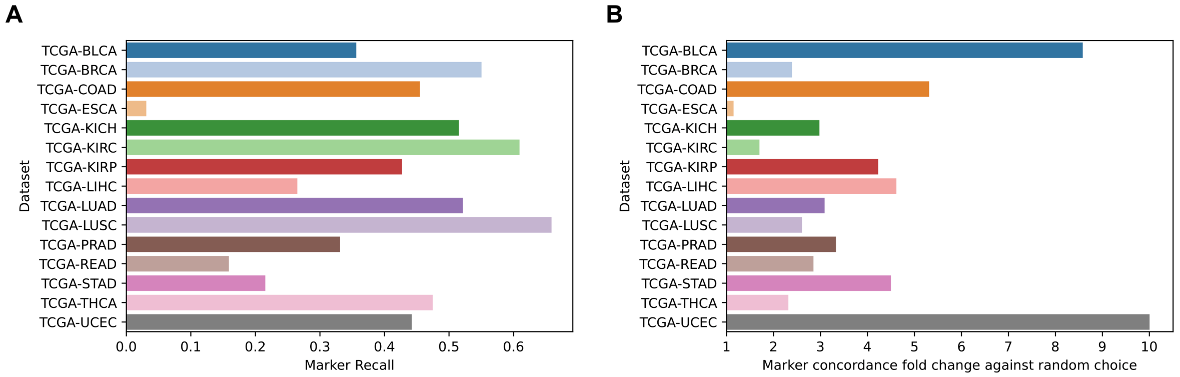

C

TCGA Cancer	Reference GTEx tissue
TCGA-BLCA	Bladder
TCGA-BRCA	Breast
TCGA-COAD	Colon
TCGA-ESCA	Esophagus
TCGA-KICH	Kidney
TCGA-KIRC	Kidney
TCGA-KIRP	Kidney
TCGA-LIHC	Liver
TCGA-LUAD	Lung
TCGA-LUSC	Lung
TCGA-PRAD	Prostate
TCGA-READ	Colon
TCGA-STAD	Stomach
TCGA-THCA	Thyroid
TCGA-UCEC	Uterus
TCGA-HNSC	N/A

Figure S23 : Using GTEx normal tissues instead of TCGA normal tissue, related to STAR Methods

The predictive power of per-cancers markers was confirmed as we accurately separated tumours from normal GTEx tissues (instead of TCGA normal tissues) using a machine learning classifier **A.** Recall statistics, the fraction of shared markers between markers identified from TCGA normal or GTEx normal. **B.** The Fold Change analysis corresponding to the observed number of shared markers against the expected value if they were chosen at random. All fold changes are statistically significant (hypergeometric test, p<1e-300) except for TCGA-ESCA. **C**. Table showing GTEx normal tissue to TCGA cancer matching.

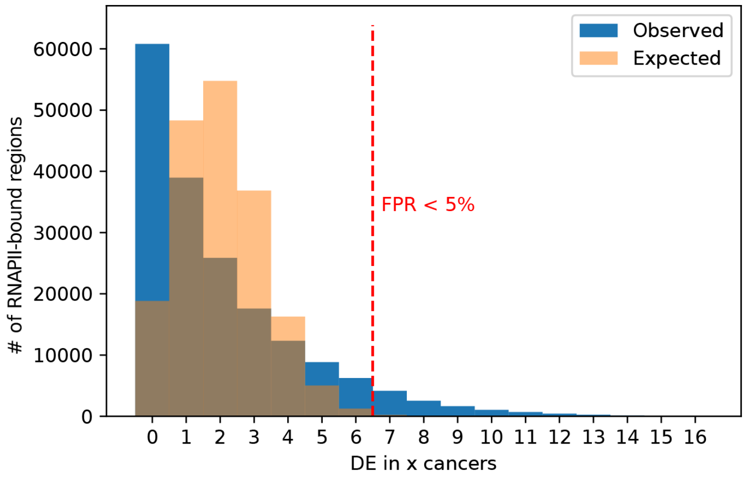

Figure S24 : Distribution of differentially expressed RNAPII-bound regions in cancers, related to STAR Methods

Distributions of the number of cancers of RNAPII-bound region is DE in, as observed in the TCGA dataset, and by cancer-wise random permutations. Red dashed line indicates the threshold at which less than 5% of observed DE RNAPII-bound regions are DE in more cancer than expected by chance.
